# The nuclear Argonaute HRDE-1 directs target gene re-localization and shuttles to nuage to promote small RNA-mediated inherited silencing

**DOI:** 10.1016/j.celrep.2023.112408

**Published:** 2023-04-20

**Authors:** Yue-He Ding, Humberto J. Ochoa, Takao Ishidate, Masaki Shirayama, Craig C. Mello

**Affiliations:** 1 RNA Therapeutics Institute, University of Massachusetts Medical School, Worcester, MA 01605, USA; 2 Howard Hughes Medical Institute, Worcester, MA 01605, USA; 3 Lead contact

## Abstract

Argonaute/small RNA pathways and heterochromatin work together to propagate transgenerational gene silencing, but the mechanisms behind their interaction are not well understood. Here, we show that induction of heterochromatin silencing in *C. elegans* by RNAi or by artificially tethering pathway components to target RNA causes co-localization of target alleles in pachytene nuclei. Tethering the nuclear Argonaute WAGO-9/HRDE-1 induces heterochromatin formation and independently induces small RNA amplification. Consistent with this finding, HRDE-1, while predominantly nuclear, also localizes to peri-nuclear nuage domains, where amplification is thought to occur. Tethering a heterochromatin-silencing factor, NRDE-2, induces heterochromatin formation, which subsequently causes *de novo* synthesis of HRDE-1 guide RNAs. HRDE-1 then acts to further amplify small RNAs that load on downstream Argonautes. These findings suggest that HRDE-1 plays a dual role, acting upstream to initiate heterochromatin silencing and downstream to stimulate a new cycle of small RNA amplification, thus establishing a self-enforcing mechanism that propagates gene silencing to future generations.

## In brief

Ding and colleagues investigate inherited silencing in *C. elegans*. They demonstrate that the nuclear Argonaute HRDE-1 induces subnuclear-co-localization of target genes in heterochromatin. Heterochromatin formation subsequently triggers *de novo* HRDE-1 guide RNA loading. Finally, HRDE-1 enters nuage and activates small RNA amplification. Thus, HRDE-1 effects multiple steps of a self-enforcing transgenerational silencing process.

## Graphical Abstract

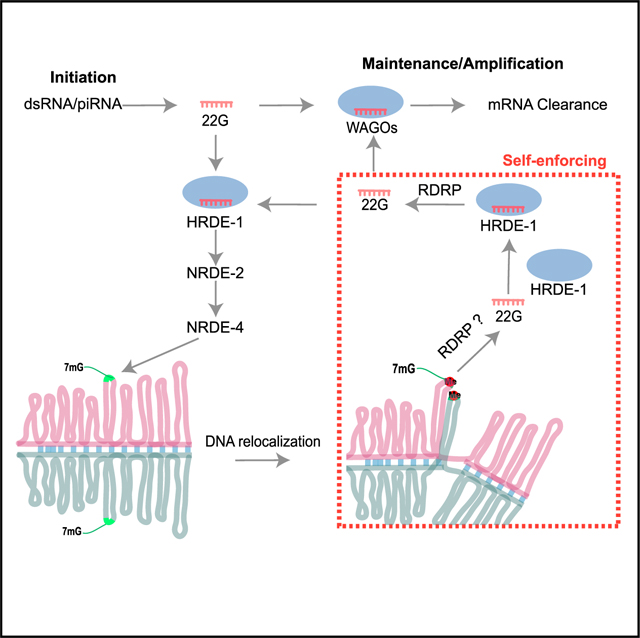

## INTRODUCTION

In many animal germlines, small RNA/Argonaute pathways function transgenerationally to install and re-inforce chromatin silencing essential for fertility. For example, in flies, worms, and mammals, members of the PIWI Argonaute family engage genomically encoded small RNAs termed PIWI-interacting RNAs (piRNAs) that silence transposons to maintain genome integrity.^1–6^ Although the details differ, all transgenerational small RNA silencing pathways studied to date require amplification and engagement of secondary Argonautes.^7^ Many of the components of the amplification machinery localize prominently in peri-nuclear non-membranous organelles called nuage. However, how the amplification system in nuage communicates with and drives the nuclear events during the initiation and maintenance of transgenerational silencing is not well understood.

In *C. elegans*, transgenerational silencing can be initiated by the PIWI pathway, by the canonical double-stranded RNA (dsRNA)-induced RNAi pathway, or by intronless mRNA.^8–11^ Inherited silencing is maintained by a family of related downstream worm-specific Argonautes (WAGO Argonautes) guided by small RNAs (22G-RNAs) produced by cellular RNA-dependent RNA polymerase. Once established, inherited silencing can be propagated independently of the initiating cues via continuous cycles of WAGO 22G-RNA amplification and transmission of the WAGO Argonautes and their small RNA co-factors to progeny.^8,12–14^

The nuclear WAGO Argonaute, HRDE-1/WAGO-9, plays a central role in transgenerational silencing in *C. elegans*.^15,16^ HRDE-1 is thought to engage nascent transcripts at target loci to induce heterochromatin and transcriptional silencing through the nuclear RNAi pathway.^15,17^ HRDE-1 promotes the transgenerational silencing of many genes^18^ and is thought to do so by recruiting chromatin remodeling factors, including the nucleosome remodeling and deacetylase complex (NuRD) and histone methyltransferases (e.g., MET-2, SET-25, SET-32).^9,18,19^ The nuclear RNAi pathway is also required for the spreading of secondary small RNAs from piRNA target sites.^14,20^

Transgenerational silencing requires a series of events that are thought to occur in the nuage, nucleus, and cytoplasm. Because all of these events are essential for the cycle of inherited silencing, their order has been difficult to determine. For example, it is not known whether the nuclear Argonaute HRDE-1 directly triggers RdRP recruitment and amplification of small RNAs or whether it must first induce heterochromatin at its targets to elicit small RNA amplification. Here, we use the phage lambda N (λN)-boxB tethering system^21–25^ to recruit—i.e., tether—HRDE-1 or the nuclear silencing factor NRDE-2 to a reporter mRNA. In principle, tethering enables initiation of silencing in the absence of upstream initiators such as piRNAs or dsRNA and, with appropriate genetic tests, can be used to order events in the pathway. We show that tethering either HRDE-1 or NRDE-2 can induce a complete silencing response, including small RNA amplification and transgenerational silencing that persists even after the λN-fusion protein is crossed from the strain. Tethering NRDE-2 initiates chromatin silencing through *nrde-4* and independently of *hrde-1* but requires *hrde-1* for small RNA amplification. By contrast, tethering HRDE-1 stimulates chromatin silencing through NRDE-2 and NRDE-4 but can elicit small RNA amplification independently of both these chromatin-silencing factors. Mutations that block HRDE-1 from binding small RNA disarm silencing and cause HRDE-1 to become cytoplasmic, but tethering HRDE-1 in these mutants nevertheless initiates a strong silencing response that requires small RNA amplification proximal to the tether site. The small RNA amplification machinery is recruited to the tether site by sequences in the N-terminal half of HRDE-1 (the N-terminal domain [NTD]). Like full-length HRDE-1 protein, HRDE-1 NTD co-localizes with MUT-16 in Mutator foci, subdomains of cytoplasmic nuage where the small RNA amplification machinery resides.^26^ Our findings suggest that HRDE-1 lies at a nexus in the silencing pathway, shuttling from the nucleus to the nuage and back, to coordinate the nuclear and cytoplasmic events of transgenerational silencing.

## RESULTS

### HRDE-1 and NRDE-2 tethering induce transgenerational silencing

**Figure 1.**
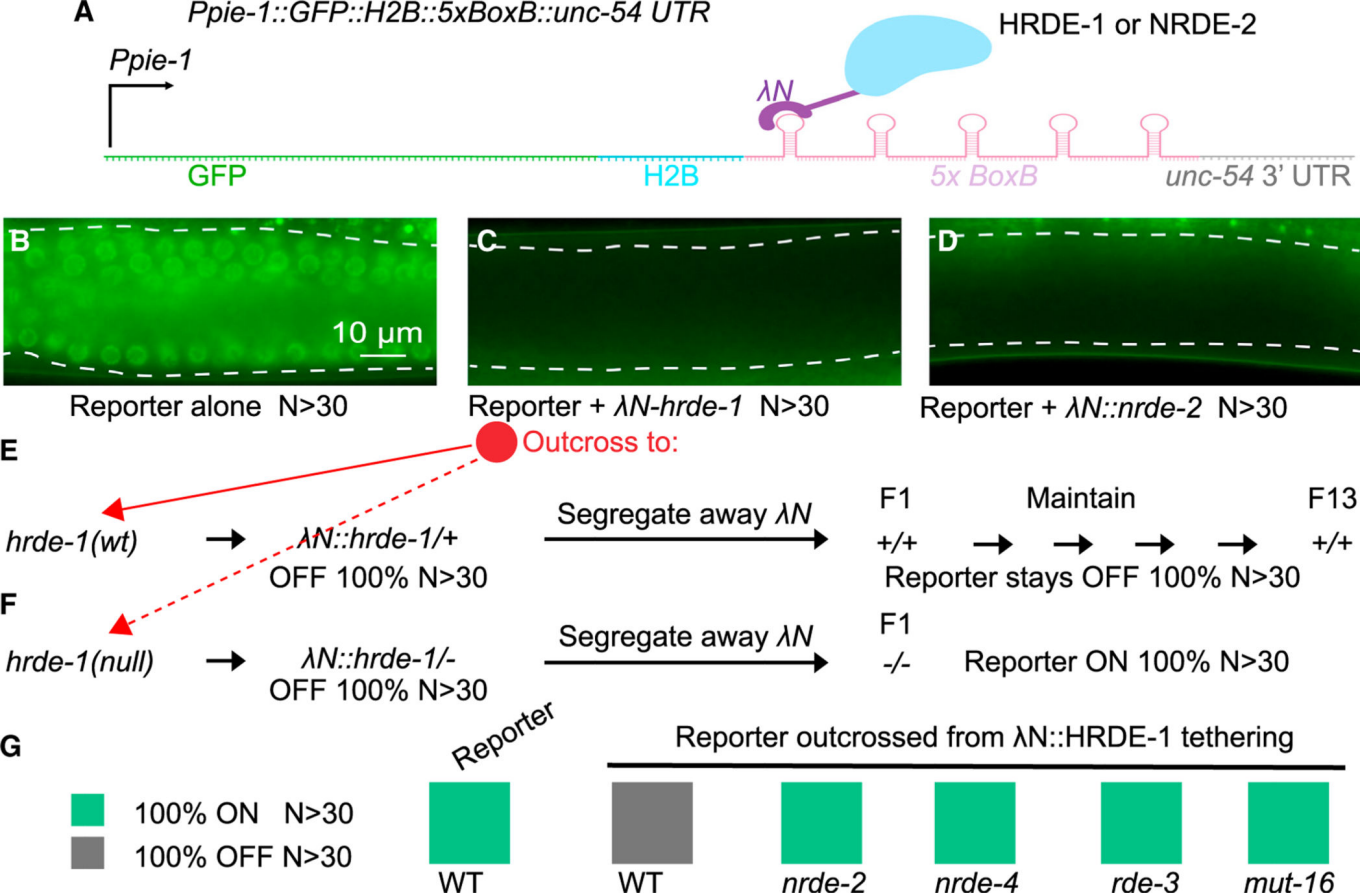
HRDE-1 tethering caused reporter silencing and generated the silencing memory (A) Scheme of λN-BoxB tethering system. A sequence encoding five BoxB hairpins (*5xBoxB*) was inserted immediately after the coding region of the *GFP::his-58(H2B)* transgene and before the *unc-54* 3′ UTR. The reporter is driven by the *pie-1* promoter (*Ppie-1*). The BoxB sites recruit λN::HRDE-1 or λN::NRDE-2 fusion proteins, thereby tethering HRDE-1 or NRDE-2 to the reporter RNA. (B) Representative fluorescence image of a syncytial germline (outlined by dashed lines) in the absence of tethering. The image represents 100% of worms scored, N > 30. (C) Representative fluorescence image in the presence of HRDE-1 tethering. The image represents 100% of worms scored, N > 30. (D) Representative fluorescence image in the presence of NRDE-2 tethering. The image represents 100% of worms scored, N > 30. (E and F) Analysis of inherited silencing triggered by λN::HRDE-1 tethering. After outcross to hrde-1 wild type (E) or *hrde-1* null (F), reporter worms were scored for *gfp* expression for 13 generations after segregating away the *λN::hrde-1* allele. The percentage of GFP+ (ON) or GFP– (OFF) worms is indicated, N > 30 worms scored in each generation. (G) Color chart showing genetic requirements of inherited silencing triggered by λN::HRDE-1 tethering. The *λN::hrde-1; reporter* worms were crossed to the indicated mutants. After segregating away *λN::hrde-1*, reporter worms homozygous for the indicated mutations were scored for GFP expression: ON or OFF, as indicated. N > 30 worms scored for each genotype.

To order events in inherited silencing, we sought to uncouple initiation and maintenance of silencing. To do this, we used the phage λN-boxB tethering system to recruit nuclear silencing factors HRDE-1 or NRDE-2 to a target reporter that is robustly expressed in the germline (Figure 1A). We hypothesized that if artificial recruitment of a silencing factor mimics a physiological event, then it should elicit a silencing response that is independent of upstream factors but depends on known downstream factors. For example, directly tethering a chromatin factor should, in principle, induce silencing without requiring machinery necessary to amplify the small RNAs that would normally guide the chromatin silencing machinery to the appropriate targets.

**Figure 2.**
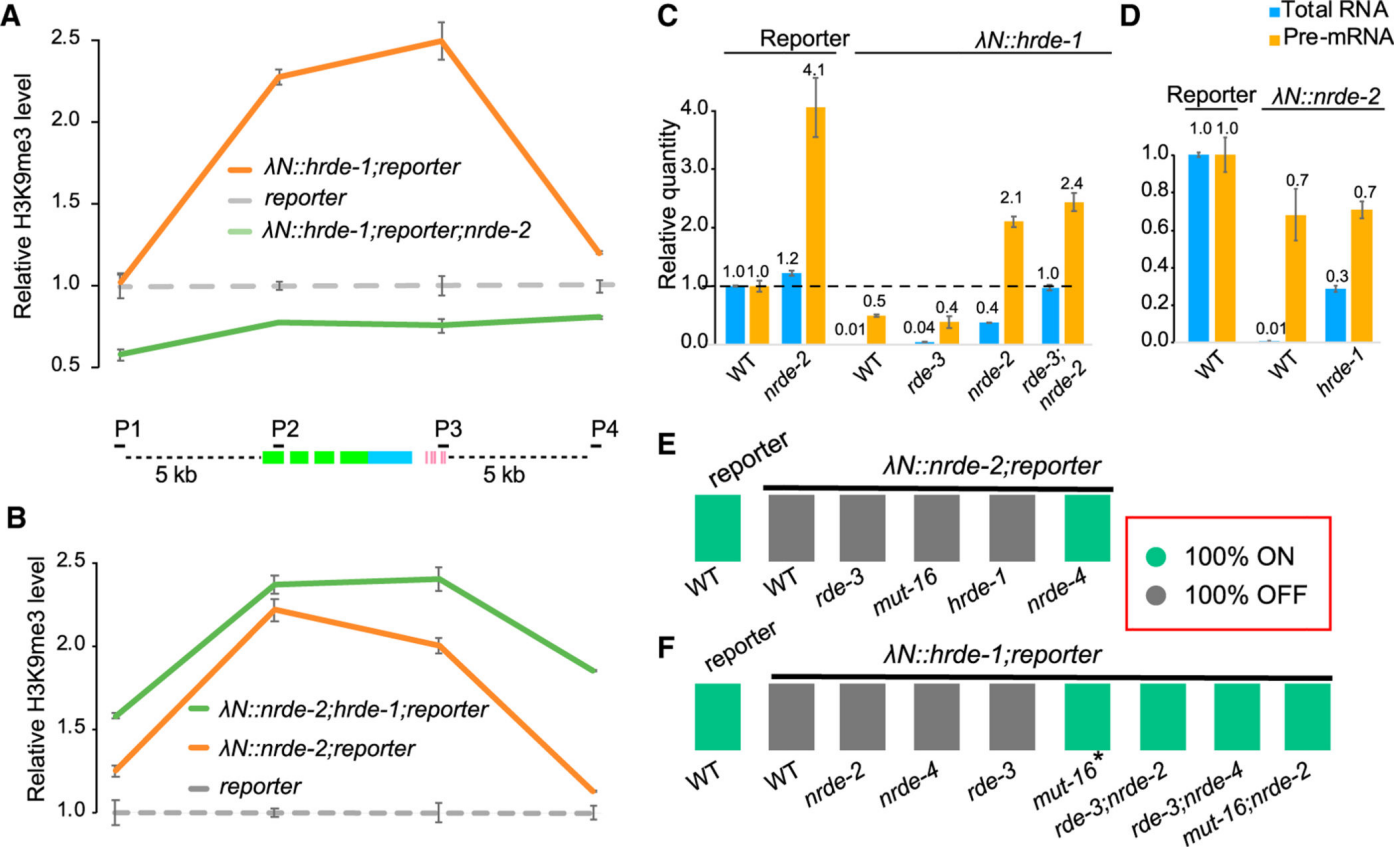
HRDE-1 and NRDE-2 tethering induce heterochromatin formation (A and B) Quantification of H3K9me3 levels near the reporter in the presence or absence of HRDE-1 or NRDE-2 tethering, as determined by chromatin immunoprecipitation (ChIP)-qPCR. P1 and P4 primer sets analyze sequences 5 kb upstream or downstream of the reporter, and P2 and P3 analyze sequences within the reporter, as indicated in the schematic. All quantities were normalized to the level of P1 in *reporter* control samples. Error bars show the standard deviation from the mean. (C and D) Bar graphs showing the quantification of reporter RNA and pre-mRNA levels in response to HRDE-1 or NRDE-2 tethering, as determined by qPCR. The average quantities relative to wild type (WT) are indicated. Error bars show the standard deviation from the mean. (E and F) Color chart showing the genetic requirements of silencing in the presence of λN::NRDE-2 or λN::HRDE-1. Reporter worms homozygous for the indicated mutations were scored for GFP expression: ON or OFF, as indicated. N > 30 worms scored for each genotype. *GFP is ON, but signal is weak (see Figure S2H).

Using CRISPR, we inserted an in-frame λN coding sequence at the 5′ end of the endogenous *hrde-1* or *nrde-2* loci (see STAR Methods). Both fusion genes were fully functional, based on their ability to mediate piRNA silencing (Figure S1A). Moreover, both strains exhibited wild-type patterns and distributions of endogenous small RNA species (Figure S1B).We then tested whether the λN fusions could induce heritable silencing of a reporter gene whose 3′ UTR contains λN-binding sites (i.e., boxB elements) (Figures 1A–1D). Both λN::HRDE-1 and λN::NRDE-2 induced silencing of the reporter beginning at the initial heterozygous generation (Figures 1C and 1D). Notably, silencing of the reporter persisted in subsequent generations after genetically segregating away the λN-fusion alleles (Figures 1E and S1C; data not shown). As expected, inherited silencing (after segregating the λN-fusion alleles) required known components of the transgenerational RNA silencing pathway, including HRDE-1, the small RNA amplification factors RDE-3/MUT-2 and MUT-16,^6,27,28^ and the nuclear silencing factors NRDE-2 and NRDE-4^9,29^ (Figures 1E–1G, S1C, and S1H; data not shown). Moreover, λN::HRDE-1 and λN::NRDE-2 tethering induced trimethylation of histone H3 lysine 9 (H3K9me3; Figures 2A and 2B) and reduced both reporter mRNA and pre-mRNA levels (Figures 2C and 2D), consistent with the role of H3K9me3 in transcriptional silencing.^15^ Thus, artificially recruiting HRDE-1 or NRDE-2 to a target locus was sufficient to initiate the full cycle of events required for inherited silencing, including small RNA amplification and heterochromatin formation.

Having established that tethering induces inherited silencing that depends genetically on known components of the RNA silencing pathway, we asked which factors were required for silencing when the tethered protein was continuously present. For example, because the λN-boxB interaction recruits HRDE-1 and NRDE-2 independently of a guide RNA, we reasoned that the small RNA amplification machinery should be unnecessary when nuclear silencing factors are tethered to the reporter. Consistent with this idea, we found that λN::NRDE-2 silenced the reporter in the absence of *rde-3*, *mut-16*, and *hrde-1* (Figures 2E, S2A–S2C) but failed to silence it in the absence of *nrde-4* (Figures 2E and S2D). These results suggest that NRDE-2 acts downstream of HRDE-1 and upstream of NRDE-4 in nuclear silencing.

In wild-type animals without tethering, inherited silencing requires nuclear chromatin silencing factors (e.g., *nrde-2* and *nrde-4*) and nuage-localized factors (e.g., *rde-3* and *mut-16*; Figure 1G), indicating that these pathways function together, possibly sequentially, to propagate inherited silencing. In contrast, when λN::HRDE-1 was tethered to the reporter, we found that leaving either pathway intact was sufficient to maintain silencing, as monitored by GFP epifluorescence. For example, silencing of the reporter GFP was maintained independently of *nrde-2*, *nrde-4*, or *rde-3* and only partly required *mut-16* activity (Figures 2F and S2E–S2H). To completely prevent silencing, it was necessary to simultaneously mutate components of both the small RNA amplification machinery (*rde-3* or *mut-16*) and components of the chromatin nuclear silencing machinery (*nrde-2* or *nrde-4*) (Figures 2F and S2E–S2K).

HRDE-1 tethering in wild-type worms reduced the unspliced pre-mRNA reporter level by 2-fold and the spliced RNA level by 100-fold, as measured by quantitative PCR (qPCR) (Figure 2C). For unknown reasons, *nrde-2* mutants exhibited a 4-fold increase in reporter pre-mRNA both with and without HRDE-1 tethering (Figure 2C) but exhibited discordant effects on spliced reporter RNA levels. Removing *nrde-2* activity in animals without tethering had little effect on spliced reporter mRNA levels (a slight 1.2-fold increase) compared with wild type, but removing *nrde-2* activity in the context of tethering caused spliced RNA levels to increase (compared with levels in wild-type HRDE-1-tethered animals), reaching levels of approximately 40% of wild-type mRNA levels. It is important to note that the qPCR assay cannot distinguish mRNA from template RNA being silenced, as template RNAs derive from spliced RNAs. Moreover, the high levels of spliced RNA in λN::HRDE-1;*nrde-2* worms correlate with a marked accumulation of reporter RNA localized in nuage (via RNA fluorescence *in situ* hybridization [FISH], shown below). Thus, the accumulated spliced RNA likely reflects template RNA engaged in amplifying the small RNA silencing signal, perhaps to compensate for the loss of heterochromatin silencing. Further study is needed to understand the effects of *nrde-2* mutants on pre-mRNA levels, such as whether increased pre-mRNA levels in *nrde-2* mutants reflect processing defects.^30^ Nevertheless, in the *nrde-2* background, HRDE-1 tethering reduces mRNA and pre-mRNA levels by 2-to 3-fold, suggesting that tethered HRDE-1 can exert effects on both mRNA and pre-mRNA levels independently of NRDE-2. Taken together, our findings suggest that HRDE-1 functions twice during inherited silencing—upstream of nuclear silencing to recruit NRDE-2 and NRDE-4 and again downstream of these factors to induce small RNA amplification and post-transcriptional clearance of mRNA. While these events likely occur sequentially and thus depend on each other during the normal course of inherited silencing,^31^ tethering HRDE-1 initiates both modes of silencing independently, either of which is sufficient to prevent reporter GFP expression.

### HRDE-1 acts downstream of NRDE-2 to promote small RNA amplification

**Figure 3.**
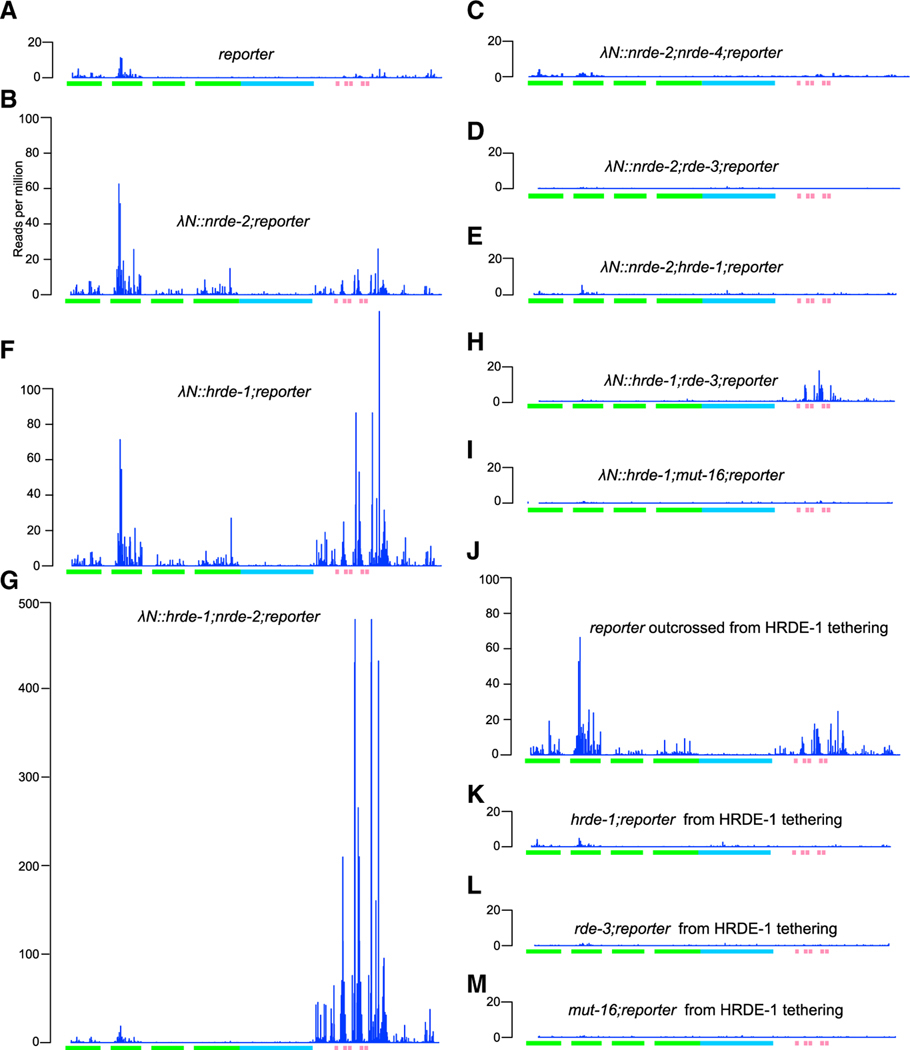
HRDE-1 and NRDE-2 tethering promote antisense small RNA production (A) Plot showing antisense small RNA reads (per million total reads) mapping to the reporter (indicated below the plot) in the absence of tethering. Only the first nucleotide is counted. Green boxes, GFP coding; blue box, H2B coding; pink boxes, BoxB hairpins. (B–E) Genetic requirements of small RNAs induced by NRDE-2 tethering. Plots showing antisense small RNA reads mapping to the reporter in the presence of λN::NRDE-2 in WT (B), *nrde-4* (C), *rde-3* (D), or *hrde-1* (E) worms. (F–I) Genetic requirements of small RNAs induced by HRDE-1 tethering. Plots showing antisense small RNA reads mapping to the reporter in the presence of λN::HRDE-1 in WT (F), *nrde-2* (G), *rde-3* (H), or *mut-16* (I) worms. (J–M) Genetic requirements of inherited small RNAs induced by HRDE-1 tethering. Plots showing antisense small RNA reads mapping to the reporter in WT (F), *nrde-2* (G), *rde-3* (H), or *mut-16* (I) worms after segregating λN::HRDE-1. Note that in (G), the y axis is compressed 50% compared with other plots to conserve space.

The above findings indicate that HRDE-1 can initiate inherited silencing independently of *nrde-2* and *nrde-4*, while NRDE-2 requires both *nrde-4* and *hrde-1*. A likely explanation for these findings is that heterochromatin silencing directed by NRDE-2 and NRDE-4 induces the *de novo* synthesis of small RNAs that engage HRDE-1 and that HRDE-1 can further amplify these small RNAs to propagate silencing to offspring. Indeed, whereas we detected very few small RNAs targeting the reporter in the absence of tethering (Figure 3A), λN::NRDE-2 induced small RNA accumulation that required *nrde-4*, *rde-3*, and *hrde-1* (Figures 3B–3E). These findings suggest that NRDE-2 tethering induces silencing and heterochromatin formation through NRDE-4 (Figures 2B and 2D) and that downstream events (e.g., heterochromatin formation itself or other NRDE-4-dependent events) act through RDE-3 and HRDE-1 to induce small RNA amplification.

λN::HRDE-1 tethering induced abundant small RNA accumulation that was independent of *nrde-2* and *nrde-4* (Figures 3F, 3G, and S3A). However, interestingly, both the distribution of small RNAs and their levels of accumulation along the target mRNA were dramatically altered in the *nrde* mutants. Small RNA levels were markedly increased adjacent to the boxB sites and were diminished on the *gfp* coding sequences (Figures 3F, 3G, S3A, and S3B). Small RNAs targeting the reporter were greatly reduced by mutations in *rde-3* and *mut-16*, as expected, (Figures 3H and 3I). Interestingly, however, a low level of small RNAs persisted directly adjacent to the boxB sites when λN::HRDE-1 was tethered in the absence of *rde-3* but not in the absence of *mut-16* (Figure 3H). This result is consistent with the observation that tethering of λN::HRDE-1 can bypass *rde-3* but cannot fully bypass *mut-16* (Figure 2F).

When outcrossed to a *hrde-1*(+) background to segregate away λN::HRDE-1, the reporter remained silent for at least 13 generations, with no change in penetrance. Moreover, we observed only a slight reduction in small RNA levels primarily in regions juxtaposed to the boxB hairpins (Figure 3J). In contrast, when outcrossed to a *hrde-1* null background, the reporter was fully de-silenced, and small RNAs were absent (Figure 3K). As expected, the maintenance of silencing, and of small RNA levels, also required *rde-3*(+) and *mut-16*(+) (Figures 3L and 3M). Taken together, these findings suggest that heterochromatin formation at the target locus induces *de novo* transcription and loading of small RNAs onto the nuclear Argonaute HRDE-1. HRDE-1, in turn, further promotes small RNA amplification and then functions again, perhaps in the next life cycle, to reinitiate heterochromatin silencing (see discussion).

### HRDE-1 guide RNA loading is not required for small RNA amplification

**Figure 4.**
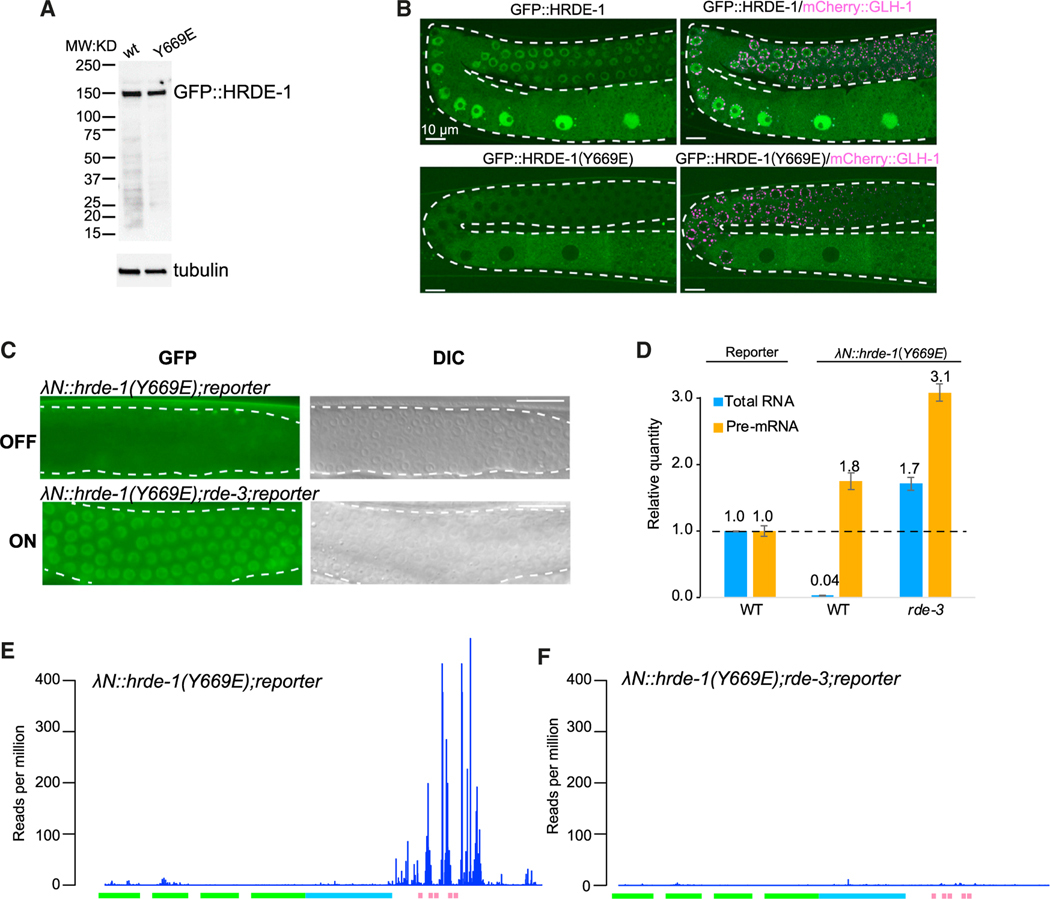
HRDE-1 guide RNA loading is not required for small RNA amplification (A) Western blot analysis to detect GFP::HRDE-1 and GFP::HRDE-1(Y669E) in worm lysates. Top panel: probed with anti-GFP antibody. GFP::HRDE-1 was indicated. Bottom panel: probed with anti-tubulin antibody as a loading control. (B) Confocal images showing the localization of GFP::HRDE-1(WT) or GFP::HRDE-1(Y669E) with mCherry::GLH-1 as P granule marker. The white dashed lines outline a gonadal arm of the germline. (C) Representative fluorescence (left panels) and differential interference contrast (DIC; right) images showing that λN::HRDE-1(Y669E) silences the BoxB reporter in WT worms (top panels, OFF) but not in *rde-3* mutant worms (bottom panels, ON). The images represent 100% of the animals scored, N > 30. (D) Bar graphs showing the quantification of reporter RNA and pre-mRNA levels in response to HRDE-1(Y669E) tethering, determined by qPCR. The average quantities relative to WT are indicated. Error bars show the standard deviation from the mean. (E and F) Plots showing antisense small RNA reads mapping to the reporter in the presence of λN::HRDE-1(Y669E) in WT (E) or *rde-3* (F) worms.

The finding that λN::HRDE-1 can direct chromatin silencing in *rde-3* and *mut-16* mutants, which are defective in small RNA amplification, suggests that the unloaded Argonaute can direct chromatin silencing when tethered. To further test this idea, we monitored silencing (1) by λN::HRDE-1 in an *hrde-2* mutant, which is defective in HRDE-1 small RNA loading^13^ and (2) by a λN::HRDE-1(Y669E) mutant, predicted by structural work to be defective in guide RNA binding (Figure S5B).^32^ In both cases, tethering completely silenced the boxB reporter as monitored by GFP fluorescence (Figure 4C and S4D) and by quantitative reverse transcription PCR (qRT-PCR) of the mRNA (Figure 4D). For unknown reasons, compromising nuclear silencing by *hrde1-(Y669E)* caused elevated pre-mRNA levels as measured by qRT-PCR (Figure 4D), similar to *nrde-2* mutants. As expected, the *hrde-1(Y669E)* mutant was defective in silencing a piRNA reporter (Figure S4A) and showed a collapse of small RNAs resembling that in *hrde-1(null)* (Figures S4B and S4C). However, in these mutant contexts, loss of *rde-3* alone was sufficient to completely de-silence the reporter (Figures S4E and 4C), suggesting that in the absence of guide RNA loading, HRDE-1 fails to engage the NRDE heterochromatin machinery. Deep sequencing revealed an abundant accumulation of *rde-3*-dependent small RNAs targeting the boxB reporter in λN::HRDE-1(Y669E) animals (Figures 4E and 4F). Notably, the pattern and levels of small RNA accumulation induced by λN::HRDE-1(Y669E) resembled those observed when wild-type λN::HRDE-1 is tethered in a *nrde-2* mutant (compare Figures 4E–3G)—i.e., resulting in increased levels of small RNAs targeting sequences adjacent to the boxB sites and reduced levels targeting GFP sequences. Taken together, these results suggest that tethering of unloaded HRDE-1 can induce local small RNA amplification and silencing but that tethered HRDE-1 must be loaded with small RNAs to induce chromatin silencing, which is in turn required for small RNA targeting to spread into the 5′ sequences of the target mRNA.

### HRDE-1 promotes small RNA amplification through its NTD

**Figure 5.**
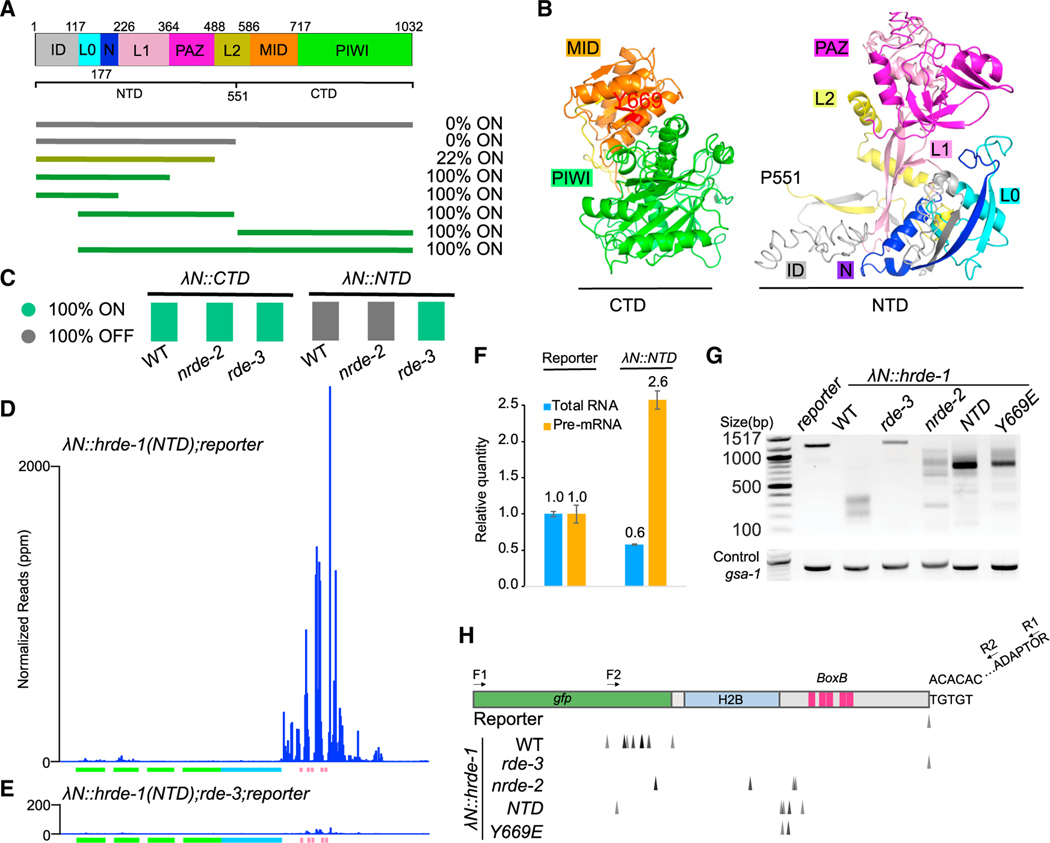
HRDE-1 N-terminal domain promotes small RNA amplification and poly-UG modification (A) Schematic showing HRDE-1 linear domain structure and truncations tested. The subdomains are color coded based on human Ago2 (Figure S5A). The percentage of GFP+ worms (ON) is indicated, N > 30 worms scored in each test. (B) Predicted three-dimensional structures of HRDE-1 N-terminal domain (NTD) and C-terminal domain (CTD). Subdomains as in (A). (C) Color chart indicating the expression (ON) or silencing (OFF) of the reporter in the presence of λN::CTD or λN::NTD and the requirement of *nrde-2* or *rde-3*. N > 30 worms scored for each genotype. (D and E) Plots showing antisense small RNA reads mapping to the reporter in the presence of λN::NTD in WT (D) or *rde-3* worms (E). (F) Bar graphs showing the quantification of reporter RNA and pre-mRNA levels in response to NTD tethering, as determined by qPCR. The average quantities relative to the control are indicated. Error bars show the standard deviation from the mean. (G and H) Analysis of poly-UG modification of reporter RNA in response to tethering in the indicated mutants. Poly-UG PCR products in (G) were cloned and sequenced to identify the precise positions of poly-UG addition (H), indicated by arrowheads. A *gsa-1*-specific PCR was used as loading control.

We next attempted to dissect functional domains of HRDE-1 required for small RNA amplification. We used CRISPR to make a series of *λN::hrde-1* truncation mutants (Figure 5A). These studies identified the N-terminal half (herein the NTD) as the minimal fragment of HRDE-1 that could fully silence the reporter. The NTD and the remaining C-terminal domain (CTD) truncations of HRDE-1 are predicted by I-TASSER^33^ to fold into self-contained globular structures, with subdomains similar to those identified in atomic resolution studies on humanAgo2^34^ (Figures 5B, S5A, and S5B). As expected, in the absence of tethering, *hrde-1(NTD)* and *hrde-1(CTD)* alleles failed to silence a piRNA sensor (Figure S4A).

Silencing by λN::NTD required *rde-3* but not *nrde-2* (Figures 5C and S5C), and deep sequencing revealed that λN::NTD induces abundant *rde-3*-dependent small RNAs targeting the boxB reporter (Figures 5D and 5E). Truncations that failed to silence the reporter did not trigger small RNA generation (Figure S5D). The small RNA pattern induced by λN::NTD resembled the patterns caused by λN::HRDE-1 in *nrde-2* mutants or by λN::HRDE-1(Y669E)—i.e., dramatically increased levels of small RNAs proximal to the boxB sites and reduced levels of small RNAs targeting GFP sequences. Interestingly, the magnitude of small RNA accumulation induced by λN::NTD at the boxB sites was ~4-fold greater than that induced by either λN::HRDE-1 in *nrde-2* mutants or by λN::HRDE-1(Y669E) (compare Figure 5D with Figures 3G and 4E). These results suggest that the NTD of HRDE-1 robustly recruits the small-RNA amplification machinery to the target and promotes silencing that is independent of the NRDE-2 nuclear silencing pathway.

### HRDE-1 tethering promotes accumulation of poly-UG-modified target fragments

During RNA silencing in worms, truncated target RNAs are converted into templates for small RNA production via the RDE-3-dependent addition of poly-UG tails.^27^ We therefore used a qPCR assay^27^ to detect poly-UG additions to reporter RNA in the absence of a λN fusion or in worms expressing λN::HRDE-1, λN::NTD, or λN::HRDE-1(Y669E) (Figures 5G and 5H). Priming from an endogenous UGUG motif in the reporter 3′ UTR serves as a control for the presence of full-length mRNA. This analysis revealed that faster-migrating, poly-UG-modified RNAs accumulated in strains where silencing was active. In wild-type λN::HRDE-1 worms, poly-UG-modified RNAs were most robustly detected at truncations within the GFP sequences (Figures 5G and 5H). As expected, only full-length mRNA was detected in *rde-3* mutants, confirming that RDE-3 is absolutely required for poly-UG RNA accumulation. Notably, mutation of *nrde-2* or tethering the NTD or Y669E mutants shifted poly-UG addition toward the 3′ end of the reporter, close to the boxB elements (Figures 5G and 5H). These results suggest that HRDE-1 tethering induces RDE-3-dependent poly-UG modification of truncation products that are generated near the tethering sites and that nuclear silencing promotes the induction of additional truncations far away from the tethering sites that likely support the 5′ spread of small RNA amplification.

To further analyze changes in target RNA caused by tethering, we used qRT-PCR. Surprisingly, whereas tethering wild-type λN::HRDE-1 reduced the reporter pre-mRNA by 50% and mRNA by 99% (Figure 2C), λN::NTD increased the reporter pre-mRNA by ~2.5-fold and reduced the mRNA by ~40% (Figure 5F). This result was surprising given that GFP fluorescence was undetectable in λN::NTD worms (Figures 5C and S5C) and suggested that the accumulating species in λN::NTD animals might reflect the accumulation of nearly full-length pUG RNA.

### Functional HRDE-1 RNA-induced silencing complex (RISC) is not required parentally for transmission of silencing to offspring

We next asked if λN::NTD can initiate inherited silencing. To do this, we first established reporter silencing by tethering λN::NTD in otherwise wild-type worms. We then crossed to a reporter strain homozygous for a *hrde-1* null allele to generate animals heterozygous for the tethering construct. Finally, we crossed these λN::NTD/null heterozygotes (either as males or hermaphrodites) to a *hrde-1*(+) reporter strain, resulting in two types of cross progeny—λN::NTD/+ or null/+ heterozygotes. Remarkably, although the λN::NTD/null parents lacked a functional HRDE-1 RISC, they nevertheless robustly transmitted silencing to the next generation (Figures S7A and S7B). As expected, HRDE-1(+) was required in the inheriting generation for silencing to occur (Buckley et al.^15^ and Figure 1F). Since the NTD fails to establish heterochromatin upon tethering and cannot directly form a RISC complex, these findings suggest that parentally established heterochromatin and HRDE-1 RISC are not required in gametes for inheritance, a finding consistent with previous work in which *hrde-1* homozygous mutant hermaphrodites were shown to transmit silencing to their heterozygous progeny.^15^ Rather, in the parental generation, the tethered NTD can stimulate amplification of small RNAs that likely engage with other Argonautes to propagate silencing to offspring (see discussion).

### HRDE-1 localizes to Mutator foci

**Figure 6.**
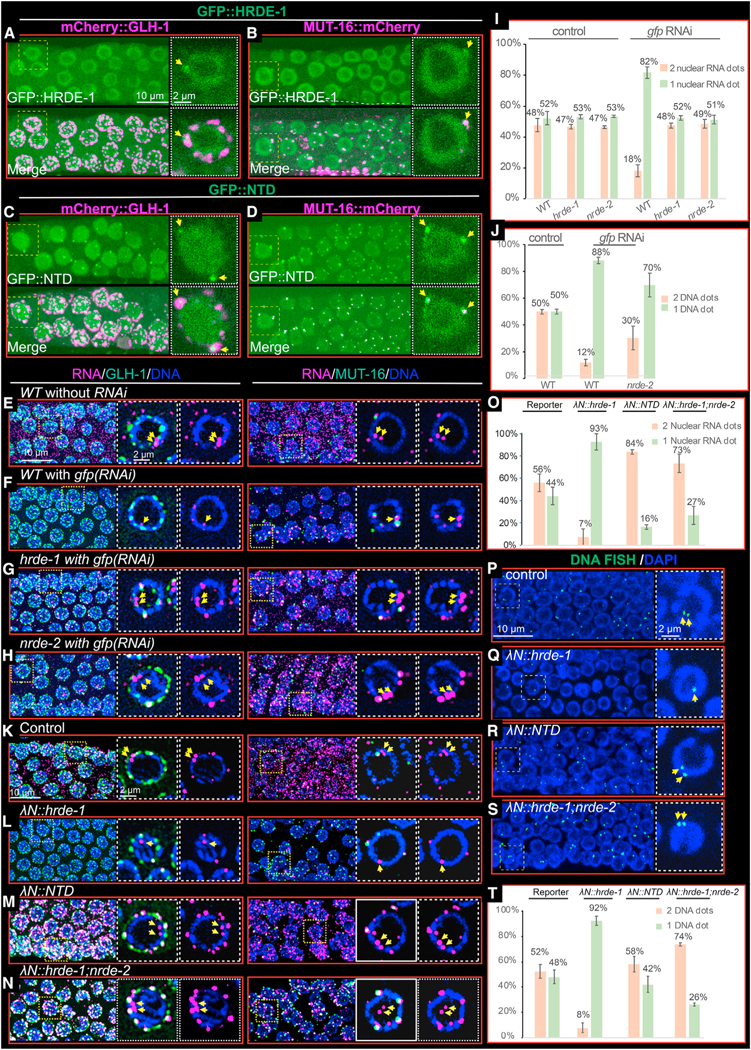
HRDE-1 localizes in Mutator foci, and HRDE-1 tethering caused peri-nuclear accumulation of reporter RNA in nuclear silencing mutants (A and B) Confocal image of live germ cells showing the co-localization of GFP::HRDE-1 with mCherry::GLH-1 (A) and MUT-16::mCherry (B). Each subpanel shows a projected view of a segment of the germline to the left, and the nucleus bounded by a dashed box is shown as a single-focal-plane image to the right. Yellow arrows point to peri-nuclear foci where HRDE-1 co-localizes with GLH-1 and MUT-16. (C and D) Confocal images of live germ cells showing the co-localization of GFP::HRDE-1(NTD) with mCherry::GLH-1 (C) and MUT-16::mCherry (D). As in (A) and (B). (E and F) Confocal images of RNA FISH experiments showing the localization of reporter RNA with mCherry::GLH-1 (left) or MUT-16::mCherry (right) in control worms (E) or in worms exposed to *gfp* RNAi (F). Magenta, RNA; green, GLH-1 or MUT-16; and blue, DAPI. Each subpanel shows a projected view of a segment of a representative germline to the left, and the nucleus bounded by a dashed box is shown as a single-focal-plane image with DNA and GLH-1 or MUT-16 signals (center) or with DNA signal only (right). Yellow arrows point to nuclear RNA foci that likely correspond to transcription sites. (G and H) As in (F) but in *hrde-1* (G) or *nrde-2* (H) mutant worms. (I) Bar graphs showing the percentage of nuclei from (E)–(H) containing one reporter RNA focus (orange) or two or more reporter RNA foci (light green). Three independent germlines were measured for each condition. Error bars show the standard deviation from the mean. (J) Bar graphs showing the percentage of nuclei from DNA FISH (Figure S6E) containing one reporter DNA focus or two or more reporter DNA foci. Similar to (I). (K–N) Confocal images of RNA FISH showing the localization of reporter RNA with mCherry::GLH-1 (left) or MUT-16::mCherry (right) in the absence (K) or presence (L–N) of HRDE-1 tethering, as indicated. Details as in (E) and (F). (O) Bar graphs showing the percentage of nuclei from (J)–(N) containing one reporter RNA focus (peach) or two or more reporter RNA foci (light green). (P–S) Confocal images of DNA FISH experiments showing the localization of reporter DNA loci in the absence (P) or presence (Q–S) of HRDE-1 tethering, as indicated. Green, DNA FISH signal; blue, DAPI. A projected view of a segment of a representative germline is shown to the left, and the nucleus bounded by a dashed box is shown as a single-focal-plane image to the right. Yellow arrows point to the nuclear DNA signals. (T) Bar graphs showing the percentage of nuclei from DNA FISH experiments in (P)–(S) containing one reporter DNA focus or two or more reporter DNA foci. Details as in (I).

HRDE-1 localization is primarily nuclear^15^; however, template formation and small RNA amplification are thought to occur in domains of peri-nuclear nuage termed Mutator foci, where several components of the small RNA amplification machinery localize.^26–28^ To examine whether HRDE-1 localizes in Mutator foci, we expressed GFP::HRDE-1 (without tethering) in worms that also express either mCherry::GLH-1, which localizes broadly within nuage, or MUT-16::mCherry, which localizes prominently in Mutator foci. GFP::HRDE-1 co-localized to a subset of peri-nuclear mCherry::GLH-1 foci, especially in association with late pachytene germ nuclei (Figures 6A and S6A). Moreover, the GFP::HRDE-1 foci only partially overlapped with mCherry::GLH-1 foci, suggesting that the HRDE-1+ foci occupy subdomains of larger GLH-1+ nuage, reminiscent of Mutator foci. Indeed, GFP::HRDE-1 foci coincided almost perfectly with MUT-16::mCherry foci (Figure 6B). Similarly, GFP::HRDE-1(NTD) co-localized with GLH-1::mCherry and mCherry::MUT-16 foci (Figures 6C, 6D, and S6B). Taken together, these findings suggest that HRDE-1 localizes via its NTD to Mutator foci, where it functions to promote small RNA amplification.

### Silencing by dsRNA or tethering causes target genes to co-localize

To understand how HRDE-1 and nuclear silencing regulate their target genes and RNAs, we performed RNA and DNA FISH studies to visualize the boxB reporter mRNA and DNA. In the absence of silencing, reporter RNA foci were detected throughout the germline cytoplasm (Figures 6E and S6C). In addition, we observed prominent RNA signals in the majority (~70%) of pachytene nuclei (most nuclei, 57%, exhibited at least two closely paired nuclear dots, while the remainder exhibited a single dot; Figures 6E and 6I). The positions of these nuclear signals adjacent to DAPI-stained chromosomes suggests that they correspond to sites of transcription on the paired sister chromatids within the axial loops of synapsed meiotic homologs. Silencing, induced either by exposure to dsRNA targeting the reporter or by tethering λN::HRDE-1, eliminated cytoplasmic reporter RNA signal and greatly reduced the nuclear signal (Figures 6F, 6L, and S6C). More than 80% of the pachytene nuclei with visible RNA signal exhibited a single nuclear focus (Figures 6F, 6L, 6I, and 6O). The changes in nuclear RNA signal induced by silencing correlated with changes in the reporter DNA FISH signal. In the absence of silencing, we observed a pair of nuclear DNA FISH signals in approximately 50% of pachytene nuclei that have visible DNA signal (Figures 6P and 6T), while in the presence of silencing, we observed a single focus of DNA FISH signal in approximately 90% of pachytene nuclei with visible DNA signal (Figures 6Q, 6J, 6T, and S6E). These results suggest that nuclear silencing mediated by HRDE-1 causes the target alleles to become merged from predominantly paired DNA FISH signals into a single focus containing all 4 silenced alleles.

### Mutations that disarm nuclear silencing cause target RNA to accumulate in nuage subdomains that resemble Mutator foci

We next examined how mutations that disarm only the nuclear silencing pathway impact RNA and DNA localization after RNAi or tethering. To do this, we performed RNA and DNA FISH on λN::NTD worms and on *nrde-2* mutants. In these mutants, where nuclear silencing is disarmed, we found that nuclear RNA and DNA FISH signals resembled the nuclear signals observed in wild-type animals in the absence of silencing: predominantly two foci of RNA and DNA FISH signals detected in each background (Figures 6M, 6N, 6I, 6J, 6O, 6T, and S6D). In contrast, however, the cytoplasmic RNA FISH signals were dramatically altered. While RNA signal was absent from the bulk cytoplasm throughout the gonad, consistent with cytoplasmic post-transcriptional silencing, we noticed pronounced accumulation of reporter RNA signals in multiple peri-nuclear foci surrounding pachytene nuclei. Co-staining experiments with GFP::GLH-1 or MUT-16::GFP revealed that these RNA foci coincide with most of the nuage subdomains that express MUT-16::GFP (Figures 6G, 6H, 6M, and 6N). The accumulation of target RNA in the MUT-16 foci required RDE-3(+) activity (Figure S6F), suggesting that these RNA signals may correspond to RdRP templates engaged in small RNA amplification.

### MUT-16 promotes the nuclear localization of GFP::HRDE-1 but not its nuage localization

**Figure 7.**
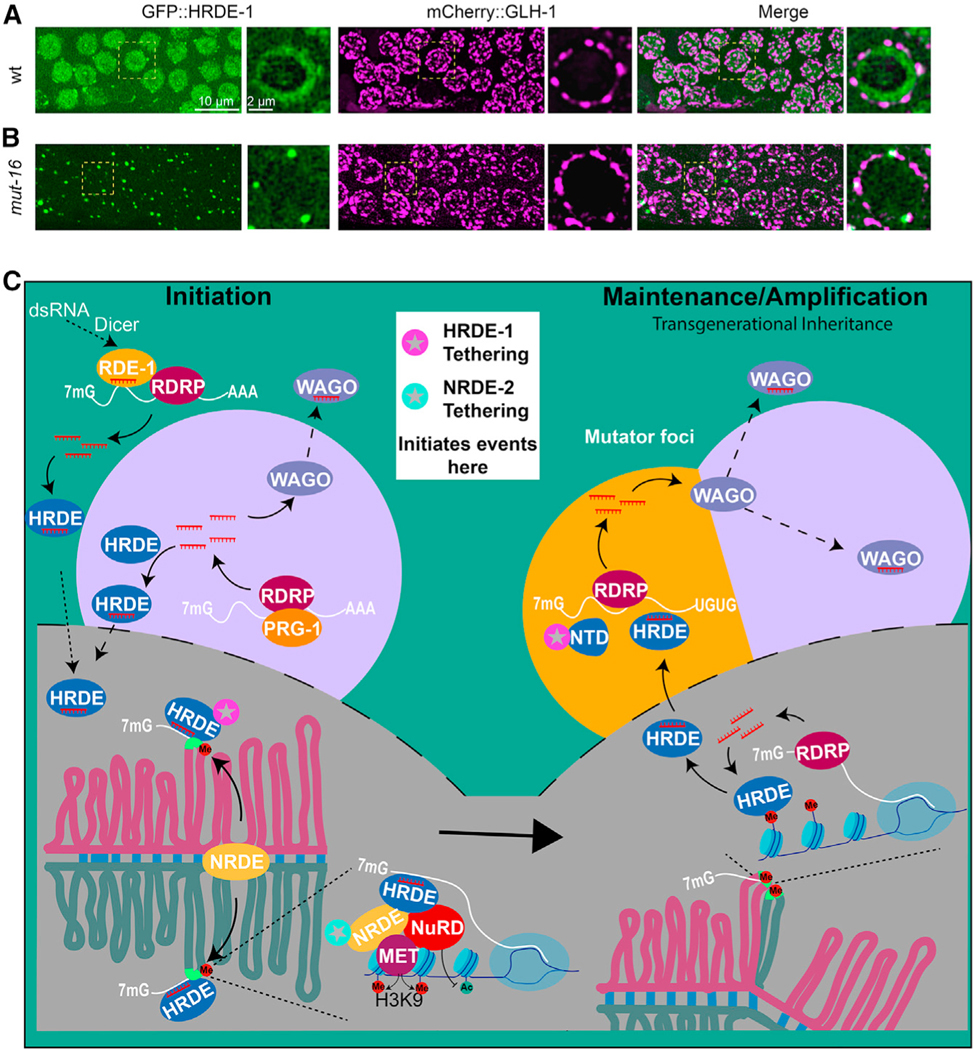
Model of HRDE-1-mediated self-enforcing mechanism (A and B) Confocal images showing the localization of GFP::HRDE-1 with mCherry::GLH-1 in WT worms (A) and *mut-16* mutants (B). Green, GFP::HRDE-1 (left); magenta, mCherry::GLH-1 (middle); merge (right). Each subpanel shows a projected image of a representative pachytene region of the germline to the left, and the nucleus bounded by a dashed box is shown as a single-focal-plane image to the right. (C) Model (see Discussion).

MUT-16 is required for the co-localization of small RNA amplification factors within Mutator foci.^26,28,35^ We therefore wondered if MUT-16 is also required for the co-localization of HRDE-1 in Mutator foci. To answer this question, we introduced a null allele of *mut-16* into worms expressing both GFP::HRDE-1 and mCherry::GLH-1. As shown previously,^24^ we found that MUT-16 activity is required for the nuclear localization of HRDE-1 (Figures 7A and 7B). MUT-16 was not, however, required for the localization of GFP::HRDE-1 to nuage (Figures 7A and 7B). The localization of GFP::HRDE-1 in nuage appeared more obvious in *mut-16* mutants, but the levels of GFP::HRDE-1 within nuage and the approximate numbers of foci appeared similar with or without *mut-16* activity (Figures 7A and 7B). Finally, the localization of MUT-16 itself to nuage was not disrupted in *hrde-1* mutants (data not shown), thus HRDE-1 and MUT-16 localize within a nuage subdomain (or domains) independently of each other.

## DISCUSSION

In many eukaryotes, the installation and maintenance of chromatin silencing is coupled to Argonaute small RNA pathways that promote transmission to offspring. Here, we have explored the role of a nuclear Argonaute HRDE-1 in coordinating transgenerational silencing in the *C. elegans* germline. In addition to its known role in directing heterochromatin silencing downstream of RNAi^13,15^ and Piwi Argonaute silencing,^8,9,14^ our tethering studies have shown that HRDE-1 is also *de novo* loaded with small RNA, downstream of heterochromatin silencing, enabling it to prime a new round of small RNA amplification within nuage (Figure 7C, model).

The nuclear silencing events that depend on HRDE-1 cause the target alleles to co-localize into a single focus of DNA FISH signal (Figures 6P–6S and S6E). Presumably, the heterochromatinized alleles within this focus are transcribed at low levels to produce template RNA that feeds transgenerational silencing; indeed, the continued expression of the target locus after heterochromatin induction is a conserved feature of co-transcriptional small RNA silencing.^36^ Consistent with this idea, the inactivation of heterochromatin silencing caused target alleles to remain separated and increased the levels of the nuclear- and nuage-localized RNA signals as measured by RNA FISH. The failure to engage nuclear silencing did not de-silence protein expression in the context of our tethering studies nor indeed in previously published studies on nuclear-silencing mutants when an RNAi trigger is present.^13,15^ Instead, our RNA FISH studies suggest that unabated transcription of the target gene feeds increased levels of target RNA localization in nuage (also noted in a recent study by Ouyang et al.^37^) and that small RNA levels also increase dramatically to compensate and silence mRNA expression. Taken together, our findings suggest that when the nuclear heterochromatin pathways are inactive, the target mRNA is silenced by a combination of cytoplasmic clearance or trapping in the P granule.

In the yeast *S. pombe*, the RNAi-induced transcriptional silencing complex (RITS), which includes an RdRP and a nuclear Argonaute AGO1p, resides in heterochromatin. A previous study showed that tethering of AGO1p to RNA via a boxB reporter system, similar to the one used here, was sufficient to recruit the RITS complex, induce small RNA amplification, and drive reporter silencing^25^.

HRDE-1 associates with NRDE-2 and components of the nucleosome re-modeling and deacetylase NuRD complex to establish heterochromatin silencing.^15,18,38^ How heterochromatin leads to *de novo* programming of HRDE-1 is nevertheless unknown. In *C. elegans*, the RdRP EGO-1 has been shown to associate with germline chromatin,^39,40^ and several of our findings would be consistent with a cycle of nuclear small RNA transcription and *de novo* HRDE-1 loading within heterochromatin. Such a mechanism could explain why tethering NRDE-2 in the absence of HRDE-1 initiates heterochromatin silencing but not small RNA amplification (Figures 2E and 3E). Perhaps after a nuclear cycle of HRDE-1 loading, the protein exits the nucleus along with nascent target/template RNA to further amplify small RNA production. Consistent with this idea, we have shown that the N-terminal half of HRDE-1 is sufficient to stimulate small RNA amplification and loading and that both the NTD and full-length HRDE-1 (as well as target RNA) localize within a specialized nuage domain known as Mutator foci.

Mutator foci accumulate poly-UG-modified templates derived from target RNA^27^ and are thought to serve in the amplification of small RNA signals that are propagated to offspring. Thus, our findings suggest that HRDE-1 shuttles out of the nucleus to nuage to promote small RNA amplification. A mutant HRDE-1 protein incapable of binding guide RNA was sufficient (when tethered) to induce silencing that transmits to offspring via either the sperm or the egg (Figures S7A and S7B). Thus, as previously reported,^15^ a functional HRDE-1 RISC is not required in gametes for transgenerational silencing but is required in offspring to renew silencing for another generation (Buckley et al.^15^ and Figure 1F). In the parental germline, Mutator foci likely serve as locations where HRDE-1 and other upstream Argonautes trigger the expansion of small RNAs that are loaded onto downstream WAGO Argonautes, including the two prominent nuage-localized Argonautes WAGO-1^8^ and WAGO-4.^41^ Consistent with this idea, silencing induced by λN::HRDE-1(Y669E) was partially dependent on *wago-1* (75% de-silenced, N = 32, and Figure S4G).

Taken together, our findings suggest that heterochromatin renews small RNA silencing (and vice versa) during each germline life cycle. For example, small RNAs guide heterochromatin formation in the zygote, and heterochromatin then propagates silencing before feeding back into the *de novo* synthesis of guide RNAs that load onto HRDE-1. HRDE-1 promotes expansion of small RNAs that are then transmitted to offspring through HRDE-1 and other WAGOs to re-establish heterochromatin. Heterochromatin then, in turn, transcribes RNA that forms templates for RdRP-dependent amplification, renewing the cycle. Consistent with these ideas, neither pathway, small RNA or heterochromatin alone, is sufficient to stably transmit silencing signals for multiple generations^8,9,13,15^ (Figures S7C–S7F). Given the similarities between the worm and yeast mechanisms—and by extension, the intriguing relationships between long non-coding RNAs and chromatin modifiers in flies and mammals^7^—feedforward RNA-chromatin circuits that amplify and maintain silencing across cell divisions or generations will likely be a common feature of gene regulation in eukaryotes.

### Limitations of the study

In this study, we use an artificial mechanism to recruit RNA silencing factors to their targets. Recruiting, factors via the λN/boxB system may elicit non-physiological mechanisms that block gene expression. For example, tethering factors to the reporter UTR could prevent proper recruitment of translation-initiation machinery or 3′ end processing factors. Transcripts that are not processed properly (for example, unspliced mRNA^11^) could trigger default recruitment of the same RNA silencing factors that mediate physiological silencing in response to bona fide Argonaute-guided silencing. To control for such possibilities, we used genetics to dissect the nature of the silencing pathways induced by tethering and found that tethering different factors elicited different genetic dependencies for silencing. For example, λN::NRDE-2 required nrde-4(+) activity for silencing but λN::HRDE-1 tethering did not. We have controlled for possible artifacts by initiating parallel studies on untethered factors and by using a combination of genetics, microscopy, and RNA-expression profiling. Together, these studies give us high confidence that tethering, in these instances, has faithfully replicated actual physiological steps in silencing.

## STAR★METHODS

### RESOURCE AVAILABILITY

#### Lead contact

Further information and requests for resources and materials should be directed to and will be fulfilled by the lead contact, Craig Mello (Craig.Mello@umassmed.edu).

#### Materials availability

All materials generated in this study are available from the lead contact without restrictions.

#### Data and code availability

Original small-RNA sequencing datasets are publicly available in NCBI under the accession number BioProject: PRJNA874806.

This study did not generate any new code, but the scripts used in the study are available from the lead contact upon request.

Any additional information required to reanalyze the data reported in this paper is available from the lead contact upon request.

### EXPERIMENTAL MODEL AND SUBJECT DETAILS

All the strains used in this study were derived from *C. elegans* Bristol N2 (CGC) and cultured on nematode growth media (NGM) plates with *E. coli* OP50^43^ or *E. coli* HT115 for RNAi experiments. Strains used in this study were generated by CRISPR-cas9 method or Cross (see Table S1 for details).

### METHOD DETAILS

#### CRISPR-Cas9 genome editing

The Cas9 ribonucleoprotein (RNP) CRISPR strategy^44^ were used to edit the genome. Plasmid pRF4 containing *rol-6* (*su-1006*) was used as co-injection marker. For short insertions like λN and deletion mutations, synthesized single-strand DNAs were used as the donor; for long insertions like GFP, mCherry, and 5xBoxB, the annealed PCR products were used instead. The gRNA and donor sequences were listed in Table S2. The BoxB reporter strain was constructed based on a single copy insertion of *Ppie-1:GFP::his-58:unc-54UTR* (WM701). The 5xBoxB sequence amplified from a previously published strain JMC002^22^ was inserted before the *unc-54* UTR.

#### Live worm fluorescent image

Young adult worms were transferred to glass slide in M9 buffer with 0.4mM Tetramisole. Epifluorescence and differential interference contrast (DIC) microscopy were performed on a Zeiss Axio Imager M2 Microscope and images were processed with ZEN Microscopy Software (Zeiss). Confocal images were taken by a Andor Dragonfly Spinning Disk confocal microscope. Confocal images were processed with Imaris Microscopy Image Analysis Software.

#### Quantifying reporter RNA using qPCR

Young adult worms were collected and washed with M9 for three times and ddH_2_O once. Total RNA was extracted with TRIZOL and treated with DNase I to remove DNA contamination. First strand cDNA was synthesized by Superscript IV with random hexamers. Quantitative PCR was performed on a Quant studio 5 Real-time PCR machine together with Fast SYBR Green Master Mix. Actin was used as internal reference (primer set S5265 and S527). Primer set of oYD826 and oYD827 were used for reporter. All primers used were listed in Table S2.

#### CHIP-qPCR

A traditional worm CHIP method^45^ was applied to the young adult worm samples. Anti H3K9me3 antibody (Upstate 07523) and CHIP grade IgA/G magnetic beads were used for the immunoprecipitation. During elution, RNase A and Protease K were used to remove RNA and proteins. For qPCR, actin was used as internal reference. All primers used were listed in Table S2.

#### Small RNA cloning and data analysis

Small RNA cloning was conducted as previously reported.^6^ Synchronized young adult worms were collected and total RNA were purified with Trizol. Two biological repeats were included for each strain. Small RNAs were enriched using a mirVana miRNA isolation kit. Homemade PIR-1 was used to remove the di or triphosphate at the 5′ to generate 5′ monophosphorylated small RNA. Adaptors of 3’ (DA35) and 5’ (DA4) were ligated to the small RNA by T4 RNA ligase 2 (NEB) and T4 ligase 1 (NEB) sequentially. Reverse transcription was performed with SuperScript III and RT primer (DA5). After PCR amplification, productions around 150 bp were separated by 12% SDS-PAGE and equally mixed. Libraries were sequenced on a NextSeq 550 sequencer with the illumina NextSeq 500/550 high output kit in 75bp single-end sequencing mod. Reads were trimmed by cutadapt and mapped using Bowtie2.^42^ For small RNAs mapped to the reporter, total reads with length longer than 16 nt were used to normalized between samples. Plots were generated by R and R studio.

### QUANTIFICATION AND STATISTICAL ANALYSIS

To determine the genes with increased or decreased antisense small RNAs (Figures S4B and S4C), small RNAs were cloned and sequenced as described above with two biological repeats for each strain. DEseq2 package in R was used to find out genes with 2-fold decrease of antisense small RNA (p value ≤ 0.05) in *hrde-1*(*null*) or *hrde-1*(*Y669E*) compared to WT.

#### Structure prediction

The 3D structure of HRDE-1 was predicted by I-TASSER online server^33^ with default setting. HRDE-1 structure was aligned with hAgo2 by PyMOL^46^ and its domains were annotated based on the alignment.

#### pUG RNA analysis

As previously reported,^27^ total RNAs were extracted with Trizol. SuperScript IV was used to generate the first strand DNA with reverse transcription primer oYD1001. A pair of outer primers (oYD998 and oYD1002) were used for the first round PCR amplification with Taq DNA polymerase. After 100-fold dilution, another round of PCR was performed with a pair of inner primers (oYD256 and oYD1003). PCR products were analyzed by 1.5% agarose gels. DNA bands were purified, cloned with TOPO TA Cloning Kit and sent for sanger sequencing. *gsa-1* served as a control for pUG PCR analysis.

#### RNA FISH

Worms at young adult stage were dissected in Happy Buffer (81mM HEPES pH 6.9, 42mM NaCl, 5mM KCl, 2mM MgCl2, 1mM EGTA) (From personal correspondence with James Priess). Dissected gonads were transferred to poly-lysine treated dish with 80 μl of Happy Buffer and fixed by adding equal volume of 5% formaldehyde in PBST (PBS+0.1% Tween 20) for 30 min. After one wash with PBST, gonads were treated with PBST-Triton (PBST+0.1% Triton) for 10 min, washed with PBST again and emerged in 70% ethanol for 30 min to overnight. Before hybridization, samples were washed with fresh wash buffer (2xSSC +10% formamide) for 5 min hybridization was performed at 37°C for 18 h to overnight in hybridization buffer (900 μl Stellaris RNA FISH Hybridization Buffer+ 100ul formamide) with 10 pmol RNA FISH probes. Samples were washed with wash buffer, once quick wash, one wash for 30 min at 37°C and two quick washes. Mounting medium with DAPI was added to preserve the signal. Confocal images were taken with an Andor Dragonfly Spinning Disk confocal microscope and processed with Fusion and Imaris.

#### DNA FISH

Same to RNA FISH, gonads were dissected, fixed and washed with PBST and treated with 70% ethanol. Then, samples were washed with wash buffer three times, one at room temperature for 5 min, one at 95°C for 3 min, and one at 60°C for 20 min. Hybridization was performed in hybridization buffer (700 μl Stellaris RNA FISH Hybridization Buffer +300 μl formamide + primary probes (final 10 pmol) + detection probe (final 10 pmol)) at 95°C for 5 min and then transferred to 37°C for 3 h to overnight. After hybridization, samples were wash with 2xSSC for 20 min at 60°C, and then 2xSSCT (2xSSC +0.3% Triton X-100) for 5 min at 60°C and another 20 min at 60°C.

After another wash with 2xSSCT for 5 min at room temperature, samples were preserved in the mounting medium with DAPI. Confocal images were taken with an Andor Dragonfly Spinning Disk confocal microscope and processed with Fusion and Imaris. Primary probes of DNA FISH were picked from the oligo lists generated by OligoMiner.^47^

#### RNAi experiments

Synchronous L1 worms of the reporter strain were plated on NGM plates for 48 h. Then the worms were collected and washed with M9. About 100 worms were plated on every IPTG plate with the *gfp* RNAi food. After 24 h, worms were dissected for the FISH experiment. RNA FISH and DNA FISH were performed as described above.

## Supplementary Material

1

## Figures and Tables

**Table:** KEY RESOURCES TABLE

REAGENT or RESOURCE	SOURCE	IDENTIFIER
Antibodies		

Anti-GFP	Cell Signaling	Cat# 2956S
Anti-Tubulin	Abcam	Cat# ab6160; RFID:AB_305328
Anti-H3K9me3	Upstate	Cat# 07523; RFID:AB_310687

Bacterial and virus strains		

*E. Coli*: OP50	CGC	WormBase: OP50
*E. Coli*: HT115	CGC	Wormbase: HT115

Chemicals, peptides, and recombinant proteins		

Alt-R^®^ S.p. Cas9 Nuclease V3	Integrated DNA Technologies (IDT)	Cat# 1081059
CRISPR-Cas9 tracrRNA	IDT	Cat# 1072534
Tetramisole hydrochloride	Sigma-Aldrich	Cat# L9756
TRI reagent (TRIZOL)	Sigma-Aldrich	Cat# T9424
DNase I	NEB	Cat# M0303S
SuperScript^™^ IV Reverse Transcriptase	Life Technologies	Cat# 18091200
Fast SYBR Green Master Mix	Life Technologies	Cat# 26616
Pierce CHIP grade IgA/G magnetic beads	Life Technologies	Cat# 26162
RNase A	Life Technologies	Cat# AM2270
Protase K	Sigma Aldrich	Cat# RPROTK-RO
SUPERase RNase Inhibitor	Invitrogen	Cat# am2696
T4 RNA Ligase 2, truncated KQ	NEB	Cat# M0373L
T4 RNA Ligase 1	NEB	Cat# M0204S
SuperScript^™^ III Reverse Transcriptase	Life Technologies	Cat# 18080085
Epredia^™^ Rite-On Frosted Thick Slide	Fisher Scientific	Cat# 1256820
Taq DNA polymerase	NEB	Cat# M0273S
Isopropyl-*β*-D-thiogalactoside (IPTG)	Sigma-Aldrich	Cat# 11411446001
Tween 20	Fisher Scientific	BP337–500
Triton X-100	Sigma-Aldrich	Cat# T8787–250mL
Saline Sodium Citrate (SSC), 20X Solution	Fisher Scientific	Cat# BP1325–1
Formaldehyde	Sigma-Aldrich	Cat# 252549
Stellaris^®^ RNA FISH Hybridization Buffer	LGC Biosearch, Genomics Ltd	Cat# SMF-HB1–10
VECTASHIELD^®^ Antifade Mounting Medium with DAPI	Vector Laboratories	Cat# H-1200–10

Critical commercial assays		

mirVana^™^ miRNA Isolation kit	Life Technologies	Cat# AM1561
KPL Detector^™^ AP Chemiluminescent Blotting Kit	SeraCare	Cat# 5910–0029
TOPO^™^ TA Cloning^™^ Kit	Life Technologies	Cat# 450641
NextSeq 500/550 High Output Kit v2.5 (75 Cycles)	Illumina, Inc.	Cat# 20024906

Deposited data		

Small RNA sequencing data	This study	NCBI (PRJNA874806)

Experimental models: Organisms/strains		

C. elegans strains	This study	Table S1

Oligonucleotides		

List of Oligonucleotides	This study	Table S2
List of RNA FISH probes	This study	Table S3
List of DNA FISH probes	This study	Table S4

Software and algorithms		

ZEN2 pro Version blue edition	ZEISS	https://www.zeiss.com/microscopy/en/products/software/zeiss-zen.html
Fusion Version 2.3.0.44	Oxford Instruments	https://fusion.help.andor.com
Microscopy Image Analysis Software: Imaris Version 9.7.1	Oxford Instruments	https://imaris.oxinst.com
ImageJ	NIH	https://imagej.net
Python3	Python.org	https://www.python.org
Cutadapt version 4.1	NBIS	https://cutadapt.readthedocs.io
Bowtie2 Version 2.4.5	Langmead et al.^42^	https://bowtie-bio.sourceforge.net/bowtie2
R Version 4.2.1	R-project.org	https://www.r-project.org
R studio Version Build 554	Posit Software	https://posit.co/download/rstudio-desktop/
DESeq2 version 1.26.0	Bioconductor	https://bioconductor.riken.jp/packages/3.0/bioc/html/DESeq2.html
I-TASSER	Yang et al.^33^	https://zhanggroup.org/I-TASSER/
PyMOL(TM) Molecular Graphics System Version 2.5.0	Schrodinger	https://pymol.org

## Highlights

- Nuclear Argonaute HRDE-1 separately induces heterochromatin and small RNA production
- HRDE-1 induces target alleles to merge into a single focus of heterochromatin
- Transcription within heterochromatin feeds *de novo* loading of HRDE-1 with small RNAs
- HRDE-1 shuttles to nuage and promotes small RNA production via its N-terminal domain
