# Supplementary material for: The nuclear Argonaute HRDE-1 directs target gene re-localization and shuttles to nuage to promote small RNA-mediated inherited silencing
Source: Cell Rep. Author manuscript; Available in PMC 2023 Aug 22. (PMC10443184; doi:10.1016/j.celrep.2023.112408)
Supplement: 1 [file NIHMS1905346-supplement-1.pdf]

Cell Reports, Volume *42*

Supplemental information

The nuclear Argonaute HRDE-1 directs target gene re-localization and shuttles to nuage to promote small RNA-mediated inherited silencing

Yue-He Ding, Humberto J. Ochoa, Takao Ishidate, Masaki Shirayama, and Craig C. Mello

Figure S1

A Expression of the piRNA reporter

Genotype	Reporter expression
wt	0% ON, N>30
hrde-1	100% ON, N>30
λN::hrde-1	0% ON, N>30
λN::nrde-2	0% ON, N>30

B 22G of WAGO targets

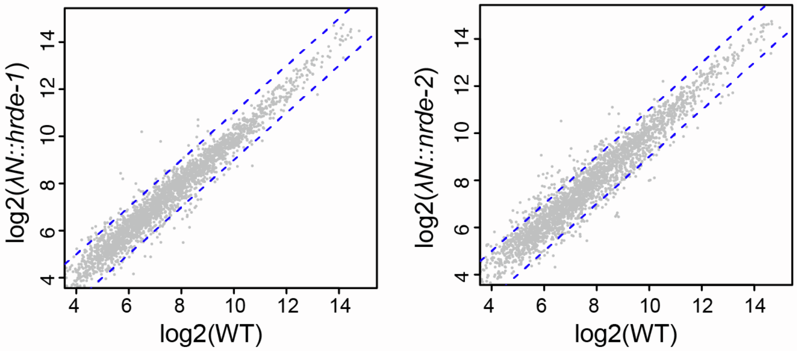

C *reporter* from HRDE-1 tethering

0% ON

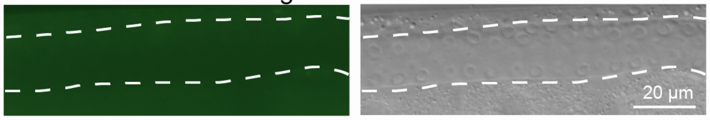

D *hrde-1;reporter* from HRDE-1 tethering

100% ON

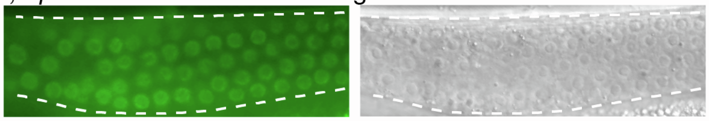

E *nrde-2;reporter* from HRDE-1 tethering

100% ON

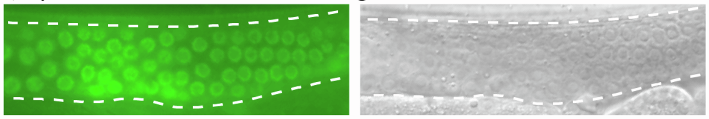

F *nrde-4;reporter* from HRDE-1 tethering

100% ON

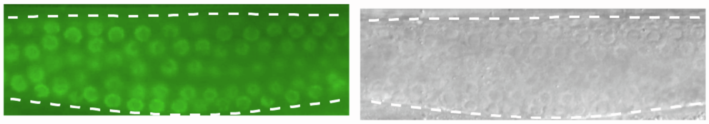

G *rde-3;reporter* from HRDE-1 tethering

100% ON

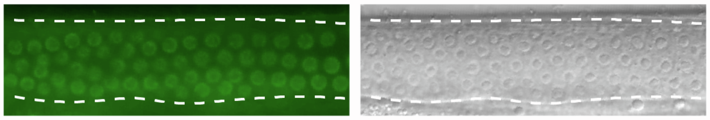

H *mut-16;reporter* from HRDE-1 tethering

100% ON

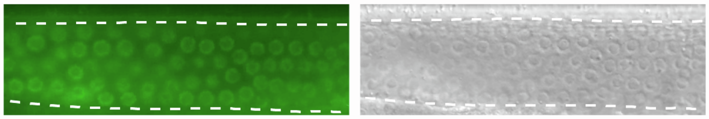

Figure S1. Expression of reporter in HRDE-1 tethering, Related to Figure 1

(A) Table shows the score of piRNA reporter expression in wild type and mutants. (B) Scatter plot of 22G against WAGO targeted genes in *λN::hrde-1* versus WT and *λN::nrde-2* versus WT. No significant differenct was observed. (C-H) Representative fluorescence (left panels) and DIC (right) images showing the requirement of inherited silencing triggered by HRDE-1 tethering (For Figure 1G). Percentage of worms with reporter ON were indicated, N>30.

Figure S2

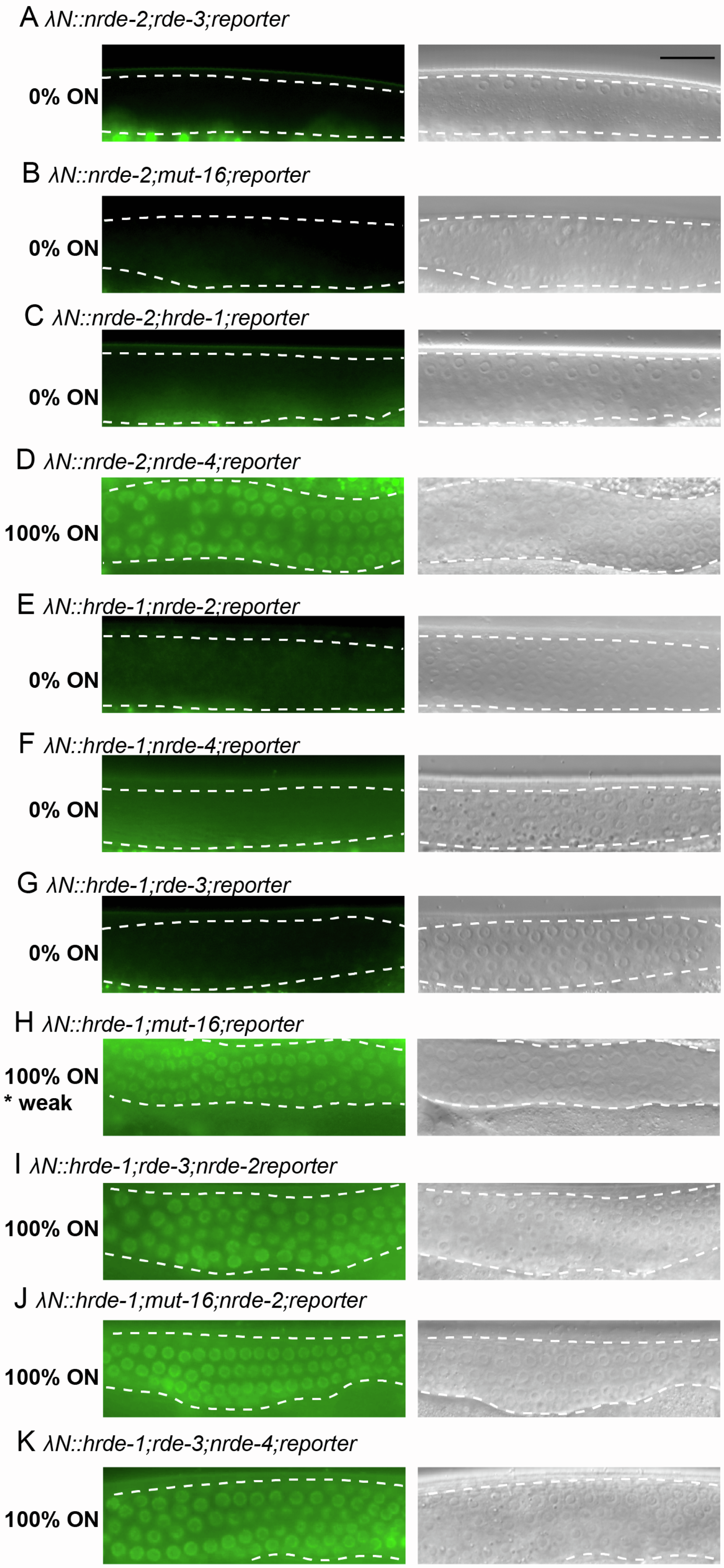

A λN::nrde-2;rde-3;reporter
0% ON
B λN::nrde-2;mut-16;reporter
0% ON
C λN::nrde-2;hrde-1;reporter
0% ON
D λN::nrde-2;nrde-4;reporter
100% ON
E λN::hrde-1;nrde-2;reporter
0% ON
F λN::hrde-1;nrde-4;reporter
0% ON
G λN::hrde-1;rde-3;reporter
0% ON
H λN::hrde-1;mut-16;reporter
100% ON
* weak
I λN::hrde-1;rde-3;nrde-2reporter
100% ON
J λN::hrde-1;mut-16;nrde-2;reporter
100% ON
K λN::hrde-1;rde-3;nrde-4;reporter
100% ON

Figure S2. Expression of reporter in HRDE-1 or NRDE-2 tethering, Related to Figure 2

(A-D) Representative fluorescence (left panels) and DIC (right) images showing the expression of boxB reporter in the presence of NRDE-2 tethering in corresponding mutants (For Figure 2E). Percentage of worms with reporter ON were indicated, N>30. (E-K) Representative fluorescence (left panels) and DIC (right) images showing the expression of boxB reporter in the presence of HRDE-1 tethering in corresponding mutants (For Figure 2F). N>30.

Figure S3

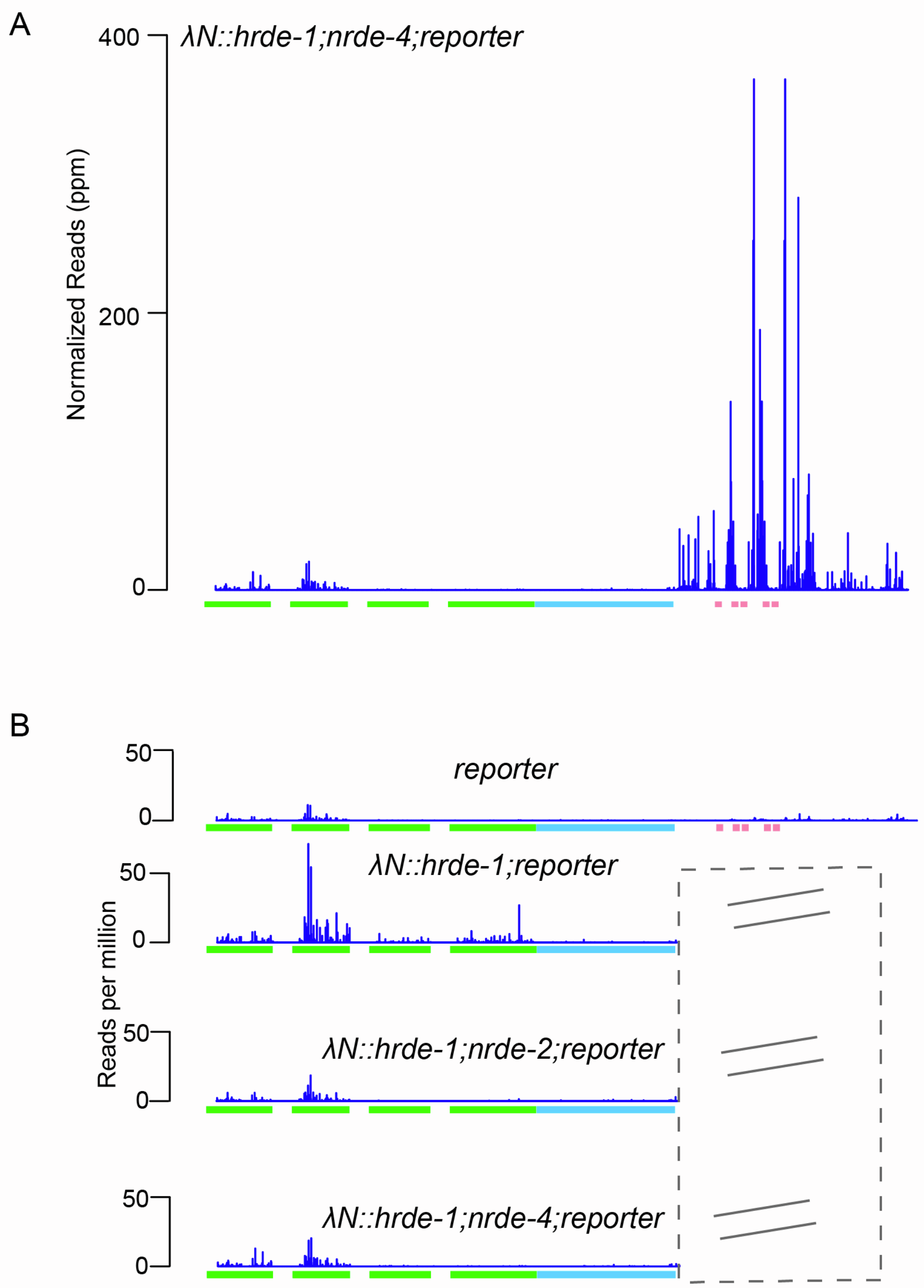

A
400
200
0
Normalized Reads (ppm)
λN::hrde-1;nrde-4;reporter
B
50
0
reporter
50
0
λN::hrde-1;reporter
Reads per million
50
0
λN::hrde-1;nrde-2;reporter
50
0
λN::hrde-1;nrde-4;reporter

Figure S3. Antisense small RNA reads mapping to the reporter, Related to Figure 3

(A) Plot showing antisense small RNA reads (per million total reads) mapping to the reporter (indicated blow the plot) in the presence of HRDE-1 tethering in the *nrde-4* mutant. (B) Plot showing antisense small RNA reads (per million total reads) mapping to the reporter in the presence of HRDE-1 tethering (corresponding to Figure 3F, 3G and S3A). Reads mapping to the BoxB region and 3' UTR were excluded.

Figure S4

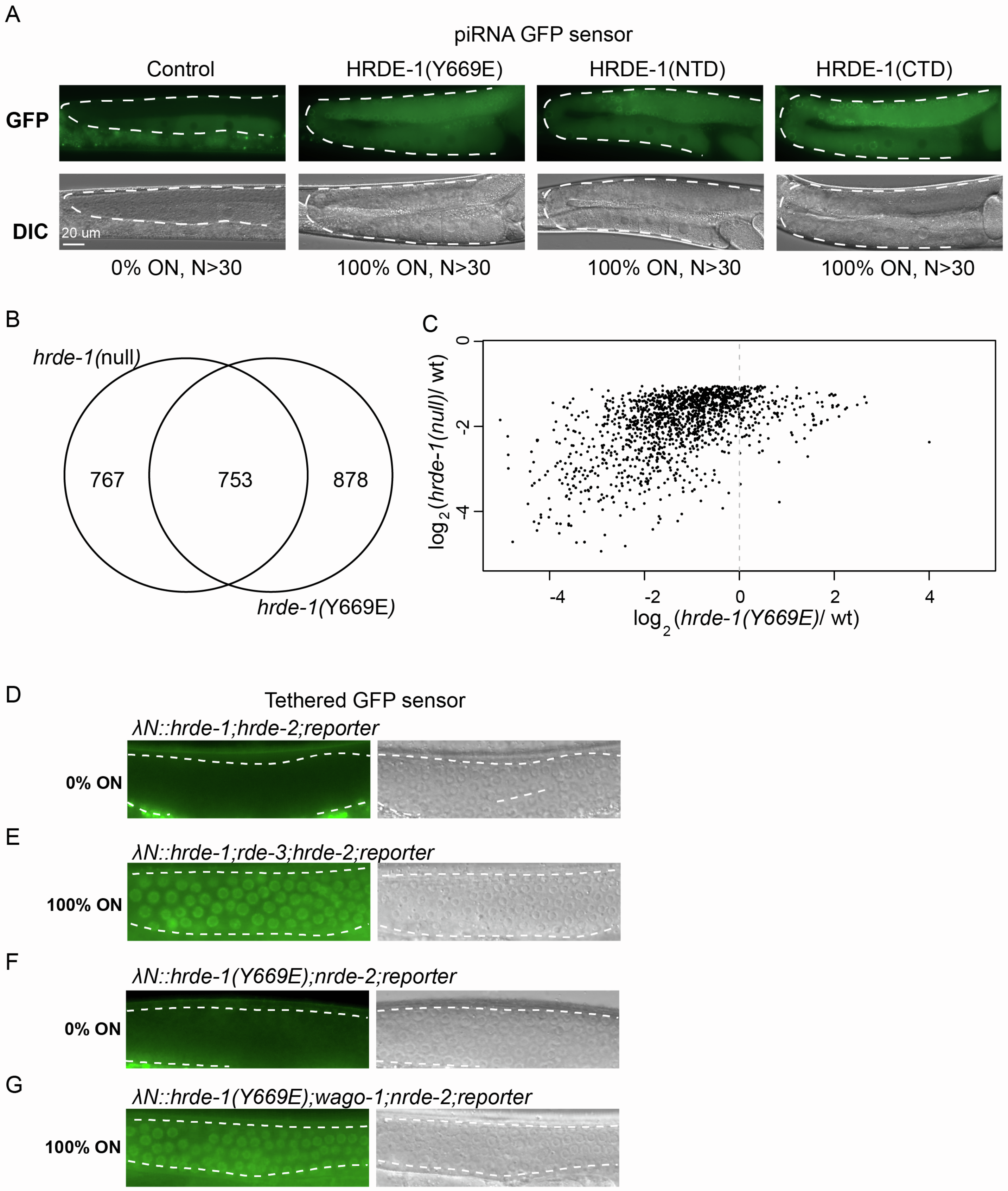
A
piRNA GFP sensor
Control
HRDE-1(Y669E)
HRDE-1(NTD)
HRDE-1(CTD)
GFP
DIC
20 um
0% ON, N>30
100% ON, N>30
100% ON, N>30
100% ON, N>30
B
hrde-1(null)
767
753
878
hrde-1(Y669E)
C
log2(hrde-1(null)/ wt)
log2(hrde-1(Y669E)/ wt)
0
-2
-4
2
4
D
Tethered GFP sensor
λN::hrde-1;hrde-2;reporter
0% ON
E
λN::hrde-1;rde-3;hrde-2;reporter
100% ON
F
λN::hrde-1(Y669E);nrde-2;reporter
0% ON
G
λN::hrde-1(Y669E);wago-1;nrde-2;reporter
100% ON

Figure S4. *hrde-1(Y669E)* is deficient in silencing the piRNA GFP sensor and small RNA biogenesis of endogenous targets, Related to Figure 4

(A) Representative fluorescence (upper panels) and DIC (lower) images showing the expression of the piRNA reporter in wild type, *hrde-1(Y669E), hrde-1(NTD)* and *hrde-1(CTD)*. (B) Ven diagram showing the overlap of genes whose antisense small RNA decreased (2-fold and P<0.05) between *hrde-1(null)* and *hrde-1(Y669E)*. (C) Plot showing the ratios of antisense small RNA level in *hrde-1(null)* versus wild type and *hrde-1(Y669E)* versus wild type. Only of genes whose small RNA level decreased (2-fold and P<0.05) in *hrde-1(null)* were plotted. (D-G) Representative fluorescence (left panels) and DIC (right) images showing the expression of boxB reporter in the presence of HRDE-1 or HRDE-1(Y669E) tethering in corresponding mutants. Percentage of worms with reporter ON were indicated, N>30.

Figure S5

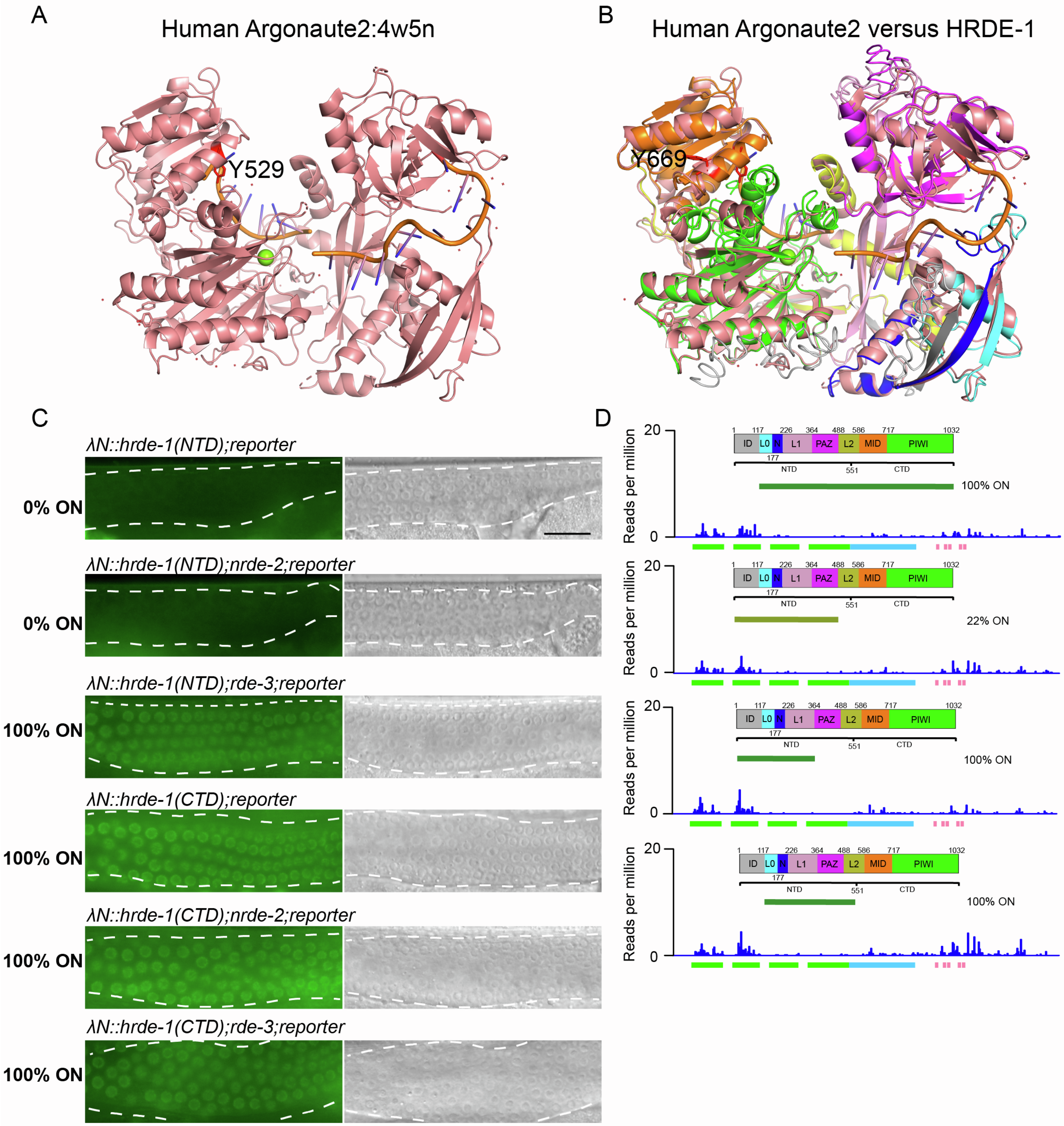
A
Human Argonaute2:4w5n
Y529
B
Human Argonaute2 versus HRDE-1
Y669
C
λN::hrde-1(NTD);reporter
0% ON
λN::hrde-1(NTD);nrde-2;reporter
0% ON
λN::hrde-1(NTD);rde-3;reporter
100% ON
λN::hrde-1(CTD);reporter
100% ON
λN::hrde-1(CTD);nrde-2;reporter
100% ON
λN::hrde-1(CTD);rde-3;reporter
100% ON
D
Reads per million
20
0
1 117 226 364 488 586 717 1032
ID L0 N L1 PAZ L2 MID PIWI
177
NTD
551
CTD
100% ON
22% ON
100% ON
100% ON

Figure S5. Tethering of HRDE-1 N-terminal domain is sufficient to trigger reporter silencing, Related to Figure 5

(A) Three-dimensional structure of human Argonaute 2 protein (PDB: 4w5n). hAgo2 is colored in pink and small RNA is colored in yellow. Image was generated by PyMOL. The phosphorylation site Y529 was indicated and colored in red. (B) Overlayed structures of hAgo2 and HRDE-1 (predicted by iTASSER). Two structures were aligned and illustrated by PyMOL. HRDE-1 domains were colored as Figure 5A. the predicted phosphorylation site Y669 of HRDE-1 was indicated and colored in red (C) Representative fluorescence (left panels) and DIC (right) images showing the expression of boxB reporter in the presence of HRDE-1 NTD or CTD tethering in wild and mutants. Scale bar is 20 um. (D) Plot showing antisense small RNA reads (per million total reads) mapping to the reporter (indicated blow the plot) in HRDE-1 truncation tethering. Scheme of the corresponding truncation is indicated in every plot.

Figure S6

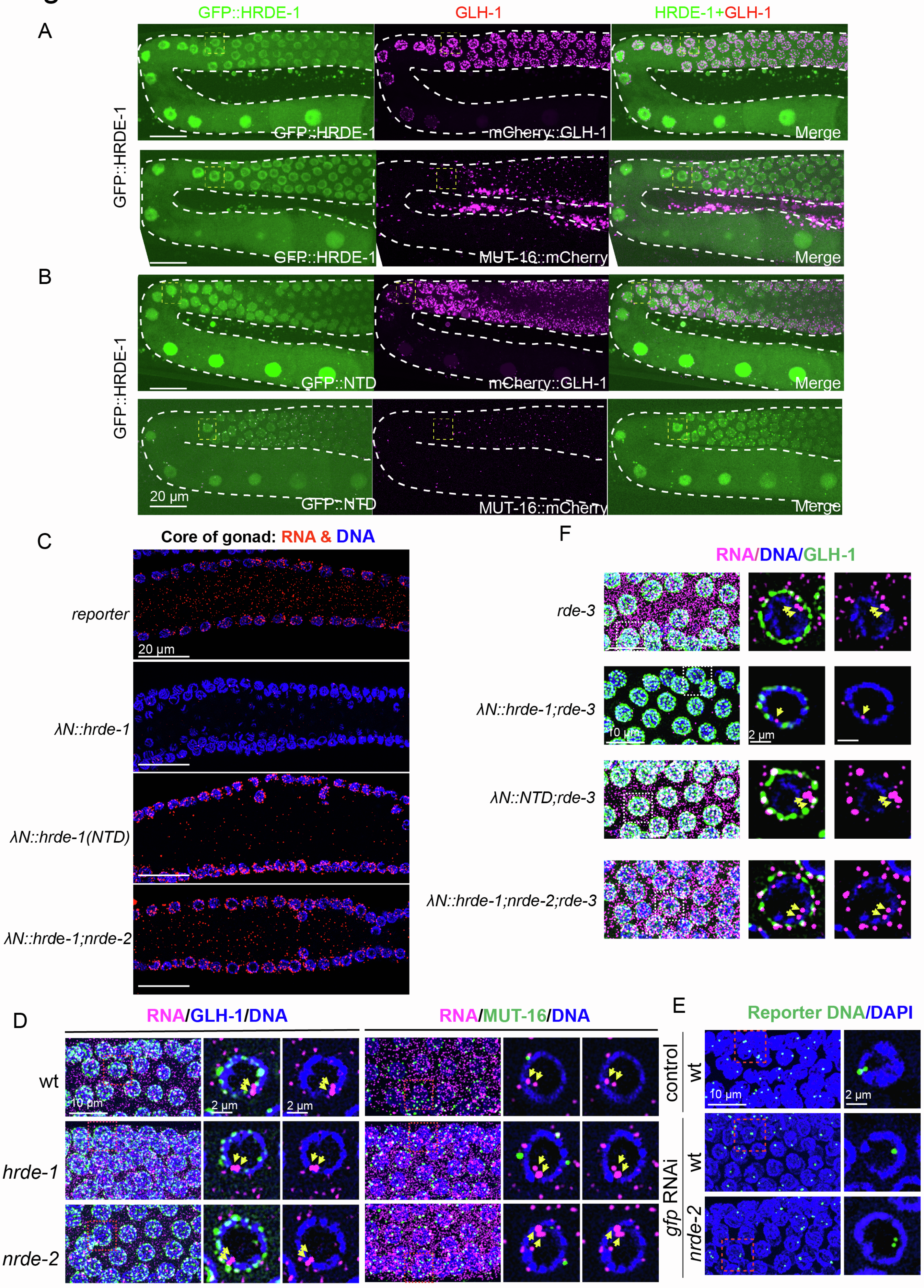
A
GFP::HRDE-1
GLH-1
HRDE-1+GLH-1
GFP::HRDE-1
GFP::HRDE-1
mCherry::GLH-1
Merge
GFP::HRDE-1
MUT-16::mCherry
Merge
B
GFP::HRDE-1
GFP::NTD
mCherry::GLH-1
Merge
GFP::NTD
MUT-16::mCherry
Merge
20 μm
C
Core of gonad: RNA & DNA
reporter
20 μm
λN::hrde-1
λN::hrde-1(NTD)
λN::hrde-1;nrde-2
F
RNA/DNA/GLH-1
rde-3
λN::hrde-1;rde-3
10 μm
2 μm
λN::NTD;rde-3
λN::hrde-1;nrde-2;rde-3
D
RNA/GLH-1/DNA
RNA/MUT-16/DNA
wt
10 μm
2 μm
2 μm
hrde-1
nrde-2
E
Reporter DNA/DAPI
control
wt
10 μm
2 μm
gfp RNAi
wt
nrde-2

Figure S6. GFP::HRDE-1 localization and FISH experiments, Related to Figure 6

(A) Confocal images showing the localization of GFP::HRDE-1 with mCherry::GLH-1 (upper panel) or MUT-16:mCherry (lower panel) corresponding to Figure 6A and 6B. Gonad is indicated inside the dashed lines. Scale bar is 20 um. (B) Confocal images showing the localization of GFP::HRDE-1(NTD) with mCherry::GLH-1 (upper panel) or MUT-16:mCherry (lower panel) corresponding to Figure 6C and 6D. (C) Confocal images showing the RNA FISH signal in the germline cytoplasm in the absence and presence of HRDE-1 tethering. Only middle slices of z stacks were projected. DNA from DAPI staining was shown in blue. (D) Confocal images of RNA FISH experiments showing the localization of reporter RNA in wild type, *hrde-1* and *nrde-2* mutants with mCherry::GLH-1 (left) or MUT-16::mCherry (right). Yellow arrows point to the nuclear RNA signals. (E) Confocal images of DNA FISH experiments showing the localization of the reporter DNA loci in the absence or presence of *gfp* RNAi. Green, DNA FISH signal; blue, DAPI. A projected view of a segment of a representative germline is shown to the left and the nucleus bounded by a dashed box is shown as a single-focal-plane image to the right. (F) Confocal images of RNA FISH experiment showing the localization of reporter RNA in the absence or presence of HRDE-1 tethering in *rde-3* mutants. Magenta, RNA FISH signal; green, mCherry::GLH-1; blue, DAPI. A projected view of a segment of a representative germline is shown to the left and the nucleus bounded by a dashed box is shown as a single-focal-plane image to the right. Yellow arrows point to the nuclear RNA signals.

Figure S7

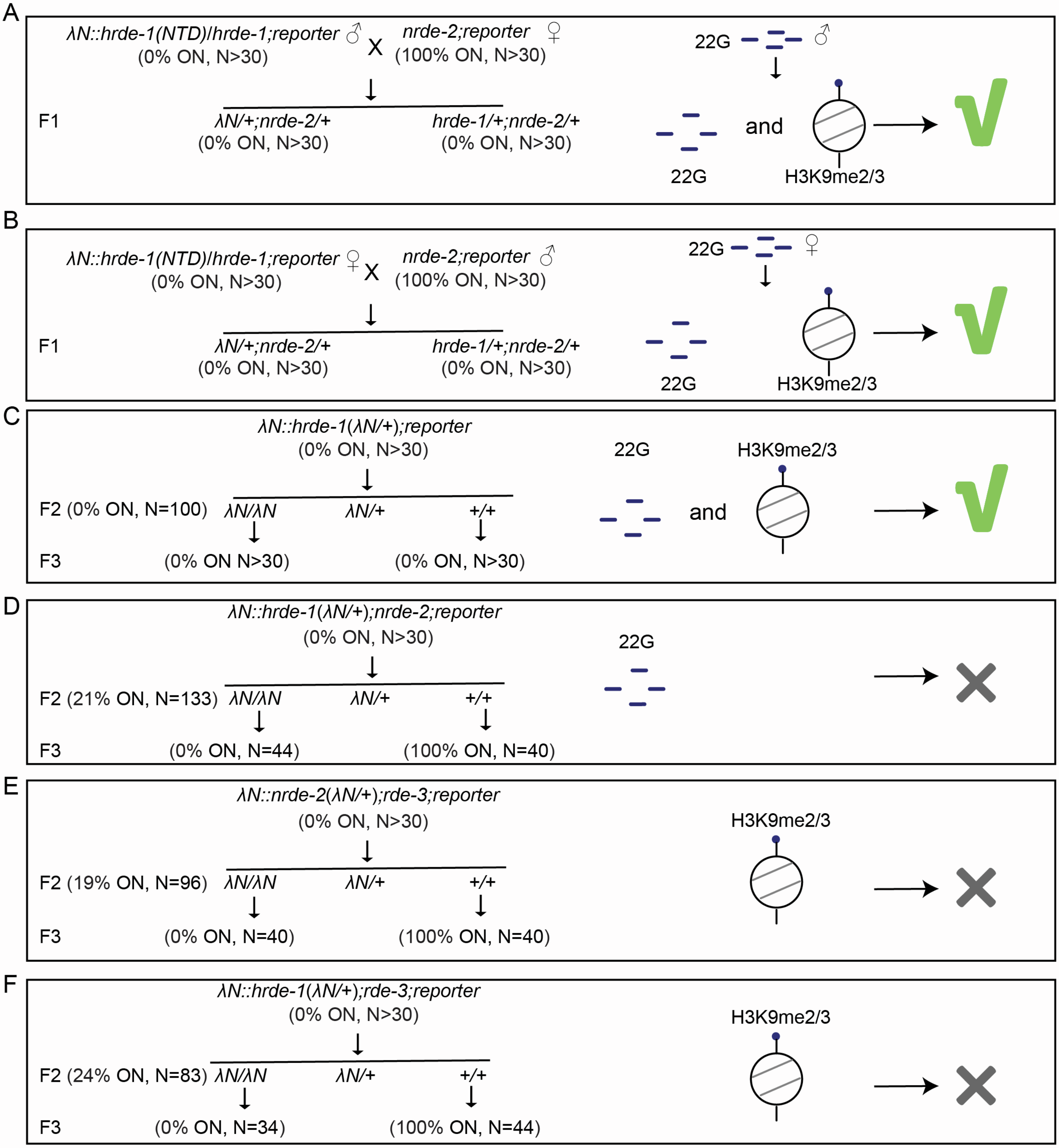
A
λN::hrde-1(NTD)/hrde-1;reporter ♂
(0% ON, N>30)
X
nrde-2;reporter ♀
(100% ON, N>30)
F1
λN/+;nrde-2/+
(0% ON, N>30)
hrde-1/+;nrde-2/+
(0% ON, N>30)
22G ♂
22G
and
H3K9me2/3
B
λN::hrde-1(NTD)/hrde-1;reporter ♀
(0% ON, N>30)
X
nrde-2;reporter ♂
(100% ON, N>30)
F1
λN/+;nrde-2/+
(0% ON, N>30)
hrde-1/+;nrde-2/+
(0% ON, N>30)
22G ♀
22G
H3K9me2/3
C
λN::hrde-1(λN/+);reporter
(0% ON, N>30)
F2 (0% ON, N=100)
λN/λN
λN/+
+/+
F3
(0% ON N>30)
(0% ON, N>30)
22G
and
H3K9me2/3
D
λN::hrde-1(λN/+);nrde-2;reporter
(0% ON, N>30)
F2 (21% ON, N=133)
λN/λN
λN/+
+/+
F3
(0% ON, N=44)
(100% ON, N=40)
22G
E
λN::nrde-2(λN/+);rde-3;reporter
(0% ON, N>30)
F2 (19% ON, N=96)
λN/λN
λN/+
+/+
F3
(0% ON, N=40)
(100% ON, N=40)
H3K9me2/3
F
λN::hrde-1(λN/+);rde-3;reporter
(0% ON, N>30)
F2 (24% ON, N=83)
λN/λN
λN/+
+/+
F3
(0% ON, N=34)
(100% ON, N=44)
H3K9me2/3

Figure S7. Genetics analysis of the role of 22G and heterochromatin in transgenerational silencing, Related to Figure 7

The mating process was shown to the left panel and the scheme of 22G and heterochromatin involved in the process to the right. Green check mark (**√)** indicates the silencing memory was transmitted to the progeny while **X** is for losing silencing memory.

Table S1, Strains used in this study, Related to STAR Methods

Strain name	Genotype	Method	Source
N2	Wild type		CGC
WM653	*neSi22 [oma-1::gfp (RNAa), cb-unc-119(+)] II; neSi10 [gfp::csr-1(RNAe), cb-unc-119(+)] IV*	MosSCI	Seth et al., 2018
WM656	*neSi22 II; hrde-1(ne4769[hrde-1Δ] III; neSi10 IV*	CRISPR	this study
WM829	*neSi22 II; ne4932[λN::hrde-1] III; neSi10 IV*	CRISPR	this study
WM830	*neSi22 II; ne4941[λN::nrde-2] III; neSi10 IV*	CRISPR	this study
WM831	*neSi22 II; ne4950[hrde-1(Y669E)] III; neSi10 IV*	CRISPR	this study
WM832	*neSi22 II; ne4949[hrde-1(NTD)] III; neSi10 IV*	CRISPR	this study
WM701	*neSi66[Ppie-1:GFP::his-58:unc-54 UTR], cb-unc-119(+)] II;unc-119(ed3) III*	MoSCI	this study
WM833	*nesi68[Ppie-1::gfp::his-58::5BoxB::unc-54 UTR, cb-unc-119(+)] II; unc-119(ed3) III*	CRISPR	this study
WM834	*nesi68 II; ne4932[λN::hrde-1] III*	CRISPR	this study
WM835	*neSi68 II* from HRDE-1 tethering	cross	this study
WM836	*rde-3(ne3370) I; neSi68 II from HRDE-1 tethering*	CRISPR	this study
WM837	*neSi68 II; hrde-1(ne4777) III from HRDE-1 tethering*	CRISPR	this study
WM838	*neSi68 II; nrde-2(ne4811)III from HRDE-1 tethering*	CRISPR	this study
WM839	*neSi68 II; nrde-4(ne4942) IV from HRDE-1 tethering*	CRISPR	this study
WM840	*mut-16(ne4810) I; neSi68 II from HRDE-1 tethering*	CRISPR	this study
WM841	*nrde-2(ne4811),nesi68 II; ne4932[λN::hrde-1] III*	CRISPR	this study
WM842	*neSi68 II; ne4932[λN::hrde-1] III; nrde-4(ne4942) IV*	CRISPR	this study
WM843	*rde-3(ne3370) I; neSi68 II; ne4932[λN::hrde-1] III*	CRISPR	this study
WM844	*mut-16(ne4810) I; neSi68 II; ne4932[λN::hrde-1] III*	CRISPR	this study
WM845	*rde-3(ne3370) I; nrde-2(ne4811), neSi68 II; ne4932[λN::hrde-1] III*	CRISPR	this study
WM846	*rde-3(ne3370) I; neSi68 II; ne4932[λN::hrde-1] III; nrde-4(ne4942) IV*	CRISPR	this study
WM847	*mut-16(ne4810) I; nrde-2(ne4811), neSi68 II; ne4932[λN::hrde-1] III*	CRISPR	this study
WM848	*neSi68 II; ne4932[λN::hrde-1] III; hrde-2(ne4943) V*	CRISPR	this study
WM849	*rde-3(ne3370) I; neSi68 II ne4932[λN::hrde-1] III; hrde-2(ne4943) V*	CRISPR	this study
WM850	*nesi68 II; ne4933[λN::hrde-1(Y669E)] III*	CRISPR	this study
WM851	*rde-3(ne3370) I;neSi68 II; ne4933[λN::hrde-1(Y669E)] III*	CRISPR	this study
WM852	*nrde-2(ne4811), nesi68 II; ne4933[λN::hrde-1(Y669E)] III*	CRISPR	this study
WM853	*wago-1(ne4821) I;nrde-2(ne4811), nesi68 II; ne4933[λN::hrde-1(Y669E)] III*	CRISPR	this study
WM854	*neSi68 II; ne4934[λN::hrde-1(NTD)] III*	CRISPR	this study
WM855	*rde-3(ne3370) I;neSi68 II; ne4934[λN::hrde-1(NTD)] III*	CRISPR	this study
WM856	*nrde-2 (ne4811), neSi68 II; ne4934[λN::hrde-1(NTD)] III*	CRISPR	this study

WM857	*neSi68 II; ne4935[λN::hrde-1(CTD)] III*	CRISPR	this study
WM858	*rde-3(ne3370) I; neSi68 II; ne4935[λN::hrde-1(CTD)] III*	CRISPR	this study
WM859	*nrde-2(ne4811), neSi68 II; ne4935[λN::hrde-1(CTD)] III*	CRISPR	this study
WM860	*neSi68 II; ne4936[λN::hrde-1(1-494)] III*	CRISPR	this study
WM861	*neSi68 II; ne4937[λN::hrde-1(1-366)] III*	CRISPR	this study
WM862	*neSi68 II; ne4938[λN::hrde-1(1-227)] III*	CRISPR	this study
WM863	*neSi68 II; ne4939[λN::hrde-1(116-551)] III*	CRISPR	this study
WM864	*neSi68 II; ne4940[λN::hrde-1(116-1032)] III*	CRISPR	this study
WM865	*ne4941[λN::nrde-2], neSi68 II*	CRISPR	this study
WM867	*rde-3(ne3370) I; ne4941[λN::nrde-2], neSi68 II*	CRISPR	this study
WM868	*mut-16(ne4810) I; ne4941[λN::nrde-2], neSi68 II*	CRISPR	this study
WM869	*ne4941[λN::nrde-2], neSi68 II; hrde-1(ne4777) III*	CRISPR	this study
WM870	*ne4941[λN::nrde-2], neSi68 II; nrde-4(ne4942) V*	cross	this study
WM871	*ne4944[mCherry::glh-1] I; ne4946[gfp::hrde-1] III*	cross	this study
WM872	*ne4944[mCherry::glh-1] I; ne4947[gfp::hrde-1(Y669E)] III*	CRISPR	this study
WM873	*ne4944[mCherry::glh-1] I; ne4948[gfp::hrde-1(NTD)] III*	CRISPR	this study
WM874	*mut-16(ne4810), ne4944[mCherry::glh-1] I; ne4946[gfp::hrde-1] III*	CRISPR	this study
WM875	*ne4945[mut-16::mCherry] I; ne4946[gfp::hrde-1] III*	CRISPR	this study
WM876	*ne4945[mut-16::mCherry] I; ne4948[gfp::hrde-1(NTD)] III*	cross	this study
WM877	*ne4944[mCherry::glh-1] I; nesi68 II*	CRISPR	this study
WM878	*ne4944[mCherry::glh-1] I; nesi68 II; ne4932[λN::hrde-1] III*	CRISPR	this study
WM879	*ne4944[mCherry::glh-1] I; nesi68; ne4934[λN::hrde-1(NTD)] III*	CRISPR	this study
WM880	*ne4944[mCherry::glh-1] I; nrde-2(ne4811), neSi68 II; ne4932[λN::hrde-1] III*	CRISPR	this study
WM881	*rde-3(ne3370), ne4944[mCherry::glh-1] I; nesi68 II*	CRISPR	this study
WM882	*rde-3(ne3370), ne4944[mCherry::glh-1] I; nesi68 II; ne4932[λN::hrde-1] III*	CRISPR	this study
WM883	*rde-3(ne3370),ne4944[mCherry::glh-1] I; nesi68; ne4934[λN::hrde-1(NTD)] III*	CRISPR	this study
WM884	*rde-3(ne3370), ne4944[mCherry::glh-1] I; nrde-2(ne4811), neSi68 II; ne4932[λN::hrde-1] III*	CRISPR	this study
WM885	*ne4945[mut-16::mCherry] I; nesi68 II*	CRISPR	this study
WM886	*ne4945[mut-16::mCherry] I; nesi68 II; ne4932[λN::hrde-1] III*	cross	this study
WM887	*ne4945[mut-16::mCherry] I; nesi68 II; ne4934[λN::hrde-1(NTD)] III*	CRISPR	this study
WM888	*ne4945[mut-16::mCherry] I; nrde-2(ne4811), neSi68 II; ne4932[λN::hrde-1] III*	cross	this study
WM889	*ne4944[mCherry::glh-1] I; neSi68 II; hrde-1(ne4777) III*	CRISPR	this study
WM890	*ne4944[mCherry::glh-1] I; nrde-2(ne4811), neSi68 II*	CRISPR	this study
WM891	*ne4945[mut-16::mCherry] I; neSi68 II; hrde-1(ne4777) III*	CRISPR	this study
WM892	*ne4945[mut-16::mCherry] I; nrde-2(ne4811), neSi68 II*	CRISPR	this study

Table S2 Oligonucleotide sequences, Related to STAR Methods

Oligo name	Type	Used for	Sequence	Source
**CRISPR guides and donors**				
oYD622	gRNA	BoxB reporter	/AlTR1/taattggacttagaagtcag/AlTR2/	IDT
oYD623	PCR primer	BoxB reporter donor primer 1	tcatcagatcgccatctcgcgcccgtgcctctgacttccaactactaa actggggattcc	IDT
oYD624	PCR primer	BoxB reporter donor primer 2	gaaagagcatgtagggatgttgaagagtaattggactccactagat caaccactttgtac	IDT
oYD252	gRNA	*λN::hrde-1*	/AlTR1/tttcttatttcagtcaaacA/AlTR2/	IDT
oYD584	ssDNA donor	*λN::hrde-1*	cgcatcgtttttcgtttcatcgtttcttatttcagtcaaacatgATGGA TGCACAAACTAGACGTAGAGAACGTCGTGCC GAGAAGCAAGCTCAGTGGAAAGCCGCTAATat ggcagacttgctcgacaaaattatgggaagttcgtcttcca	IDT
oYD560	gRNA	*λN::nrde-2*	/AlTR1/GAAACATtgttcattaagtt/AlTR2/	IDT
oYD716	ssDNA donor	*λN::nrde-2*	ttttcagggtaatatcataccaaacttaatgaacaATGGATGC ACAAACTAGACGTAGAGAACGTCGTGCCGAG AAGCAAGCTCAGTGGAAAGCCGCTAATTTTCG AGCGTATGGAAATAATGGATTGAAAAATCCAG AAA	IDT
oYD509	gRNA	*rde-3* Δ	/AlTR1/TGCAATTCATATCCCACAAG/AlTR2/	IDT
oYD510	gRNA	*rde-3* Δ	/AlTR1/TGTTTTACGATTGCACATAA/AlTR2/	IDT
oYD511	ssDNA donor	*rde-3* Δ	GGCCACTAAAAACAGTGATCTAGACGTTGCAA TTCATATCGCAATCGTAAAACAATGGGCAGCC AGTACAAAAGTTAAAG	IDT
oYD560	gRNA	*nrde-2* Δ	/AlTR1/GAAACATtgttcattaagtt/AlTR2/	IDT
oYD561	gRNA	*nrde-2* Δ	/AlTR1/aaaaagagagaaTCAACGTC/AlTR2/	IDT
oYD568	ssDNA donor	*nrde-2* Δ	ttgtgaattaagctgttttcagggtaatatcataccaaac TGAttctctctttttgtgtaactcggcctataacttttt	IDT
oYD562	gRNA	*nrde-4* Δ	/AlTR1/CCTGTTTTTTCGAATTTTAC/AlTR2/	IDT
oYD563	gRNA	*nrde-4* Δ	/AlTR1/TGTTATGTCGAAGTTGGCAA/AlTR2/	IDT
oYD572	ssDNA donor	*nrde-4* Δ	tgcacatccttgattccaaacaaggATGGATTTCACCCGT AAGGCGATTGAGGATTTCGAAAATTTGAATCT ATAAtaat	IDT
oYD735	gRNA	*mut-16* Δ	/AlTR1/ACTTTAATCGAAATACTGAT/AlTR2/	IDT
oYD736	gRNA	*mut-16* Δ	/AlTR1/GATAGTGAAATCACTTCTGA/AlTR2/	IDT
oYD737	ssDNA donor	*mut-16* Δ	AACCAACAACTTCATCGCACTTTAATCGAAATA CTTGAAGGATCCTACTCTGATGAAGATCCCGA GCAAAAGG	IDT
oYD927	gRNA	*hrde-2* Δ	/AlTR1/CCATCTTGTGAACTTCCATC/AlTR2/	IDT
oYD928	gRNA	*hrde-2* Δ	/AlTR1/GAGCAACGATGAGTACTCGC/AlTR2/	IDT
oYD929	ssDNA donor	*hrde-2* Δ	CGCAGAACAGCGACACTTTTGATTCTGGACTC CAGCCGGATGAGAGCGAGAACGACGAGAATG AATATGA	IDT
oYD545	gRNA	*wago-1* Δ	/AlTR1/GGATGCGGTTGTGGTGGATG/AlTR2/	IDT
oYD756	gRNA	*wago-1* Δ	/AlTR1/CGTTTCGGATGCTTCGAGCA/AlTR2/	IDT

oYD757	ssDNA donor	*wago-1* Δ	agaactaatattgcaatagtaataagATGTCTCCT taa TGGTCGCGGACAGAACAATCCATCTCTCGTG ATTGA	IDT
oHO1	gRNA	*hrde-1* Y669E	/AlTR1/CGACATACTCAAATACTTTG/AlTR2/	IDT
oHO2	ssDNA donor	*hrde-1* Y669E	GAAGAAGCCGGATGTTCACGACATACTCAAA **GAG**TTT**GAA**GAATCGATTGGCCTGCAAACAAT CCAATTG	IDT
oYD252	gRNA	*hrde-1* Δ	/AlTR1/tttcttatttcagtcaaacA/AlTR2/	IDT
oYD218	gRNA	*hrde-1* Δ	/AlTR1/CATTCCGAAGCGAAACTTCT/AlTR2/	IDT
oYD523	ssDNA donor	*hrde-1* Δ	cgcatcgttttttcgtttcatcgtttcttatttcagtcaaacATGTAAtc ccgagattctctcttttattgtcacgtattcaccc	IDT
oYD923	gRNA	*hrde-1* NTD(1-551)	/AlTR1/CCCTTGAACTTGATTTGAGC/AlTR2/	IDT
oYD218	gRNA	*hrde-1* NTD(1-551)	/AlTR1/CATTCCGAAGCGAAACTTCT/AlTR2/	IDT
oYD925	ssDNA donor	*hrde-1* NTD(1-551)	AGCCAATCCAAATGACTGCGAAGCTCCTCCCA CCATGGGCGTAATCCCGAGATTCTCTCTTTTA TTGTCA	IDT
oYD252	gRNA	*hrde-1* CTD(553-1032)	/AlTR1/tttcttatttcagtcaaacA/AlTR2/	IDT
oYD923	gRNA	*hrde-1* CTD(553-1032)	/AlTR1/CCCTTGAACTTGATTTGAGC/AlTR2/	IDT
oYD924	ssDNA donor	*hrde-1* CTD (553-1032)	ACTTGCTCGACAAAATTATGGAAGTTCGTCT TCCCAAATCAAGTTCAAGGGCCAGACTTACAT GCCTGA	IDT
oYD935	gRNA	*hrde-1* NTD(1-494)	/AlTR1/GATGAGCCCGACAGTACAAT/AlTR2/	IDT
oYD218	gRNA	*hrde-1* NTD(1-494)	/AlTR1/CATTCCGAAGCGAAACTTCT/AlTR2/	IDT
oYD936	ssDNA donor	*hrde-1* NTD(1-494)	AGCGGATCAAGGTCAACAAGATGAGCCCGAC AGTATGGGCGTAATCCCGAGATTCTCTCTTTT ATTGTCA	IDT
oYD937	gRNA	*hrde-1* NTD(1-366)	/AlTR1/CGAGACAGTGGTTCTTCAAA/AlTR2/	IDT
oYD218	gRNA	*hrde-1* NTD(1-366)	/AlTR1/CATTCCGAAGCGAAACTTCT/AlTR2/	IDT
oYD938	ssDNA donor	*hrde-1* NTD(1-366)	GCGACATGTACGATTCCCGTCGAGACAGTGG TTCTTGGGCGTAATCCCGAGATTCTCTCTTTT ATTGTCA	IDT
oYD223	gRNA	*hrde-1* NTD(1-227)	/AlTR1/TTCACAAAAGCTCAACAATG/AlTR2/	IDT
oYD218	gRNA	*hrde-1* NTD(1-227)	/AlTR1/CATTCCGAAGCGAAACTTCT/AlTR2/	IDT
oYD222	ssDNA donor	*hrde-1* NTD(1-227)	TGACTCGTATTATCGAAATCGTCACTTCACAA AAGCTC TAAtcccgagattctctcttttattgtcacgtattc	IDT
oYD252	gRNA	*hrde-1* NTD(116-1032)	/AlTR1/tttcttatttcagtcaaacA/AlTR2/	IDT
oYD933	gRNA	*hrde-1* NTD(116-1032)	/AlTR1/TGTAGTATTGTGCCAAGCGA/AlTR2/	IDT
oYD222	ssDNA donor	*hrde-1* NTD(116-1032)	cgttttttcgtttcatcgtttcttatttcagtcaa acATGGCAC AATACTACATCATGAGAACAGTACATGACAA	IDT
oYD252	gRNA	*gfp::hrde-1*	/AlTR1/tttcttatttcagtcaaacA/AlTR2/	IDT

oDD1	PCR primer	*gfp::hrde-1* donor primer F	/5sp9/tcgtttcatcgtttcttatttcagtcaaacatgTCCGGAG GGAGTGGA	IDT
oDD2	PCR primer	*gfp::hrde-1* donor primer R	/5sp9/GTTGGAAGACGAACTTCCCATAATTTTG TCGAGCAAGTCTGCAGAACCTCCGCCACC	IDT
cmg34	gRNA	*mCherry::glh*-1	/AlTR1/ttttctgcgaaaATGTCTGA/AlTR2/	IDT
oYD950	PCR primer	*mCherry::glh-1* donor primer F	attttctggaaaaatcttaattttctgcgaaaATGGTCTCAAAG GGTGAAG	IDT
oYD951	PCR primer	*mCherry::glh-1* donor primer R	TAGCAGCACTTTCGCTATCACTCCAACCATCA GA CAT CTTATACAATTCATCCATGCCAC	IDT
oYD968	gRNA	*mut-16::mcherry*	/AlTR1/aacaagtaaattcTTAGTTT/AlTR2/	IDT
oYD969	PCR primer	*mut-16::mCherry* donor primer F	ATTGAGCCGTATGTCGTTTTGAAAGATGATAT CCGAAAC ATGGTCTCAAAGGGTGAAG	IDT
oYD970	PCR primer	*mut-16::mCherry* donor primer R	ataaaaatggaacagtaataggaacaagtaaattcCTACTTA TACAATTCATCCATGCC	IDT
**qPCR primers**				
S526	primer	*actin* F	ggcccaatccaagagaggtatcc	IDT
S527	primer	*actin* R	gggcaacacgaagctcattgta	IDT
oYD826	primer	Reporter F	GGTGATGTTAATGGGCACAAAT	IDT
oYD827	primer	Reporter R	CTTACCCATGGAACAGGTAGTT	IDT
oYD834	primer	CHIP P1F	GCTTCTCCTTCTCTTCACATTTC	IDT
oYD835	primer	CHIP P1R	CAACTAGGCATCCGTATTTCATC	IDT
oYD826	primer	CHIP P2F	GGTGATGTTAATGGGCACAAAT	IDT
oYD827	primer	CHIP P2R	CTTACCCATGGAACAGGTAGTT	IDT
oYD830	primer	CHIP P3F	ATCTCGACTAAGTCCAACTACTAAAC	IDT
oYD831	primer	CHIP P3R	GCTGGGTCCCTCGAGATAATA	IDT
oYD832	primer	CHIP P4F	GAACTCGGAGTGAGCTTTCAT	IDT
oYD833	primer	CHIP P4R	CTCATGGATTCGGCTGACAA	IDT
**Small RNA cloning**				
DA4	RNA	5' adapter with UMI	rGrUrUrCrArGrArGrUrUrCrUrArCrArGrUrCrCrGrA rCrGrArUrCrNrNrNrCrGrArNrNrNrUrArCrNrNrN	IDT
DA35	RNA	3' adapter	/5rApp/TGGAATTCTCGGGTGCCAAGG/3ddC/	IDT
DA5	ssDNA	RT primer	CCTTGGCACCCGAGAATTCCA	IDT
**pUG amplification primers**				
oYD1001	ssDNA	RT primer	GCTATGGCTGTTCTCATGGCGTCGCCATATTC TACTTcacacacacacacaca	IDT
oYD1000	PCR primer	PCR 1F for *reporter*	ATGAGTAAAGGAGAAGAACTTTTCA	IDT
oYD1002	PCR primer	PCR 1R for *reporter* and *gsa*-1	GCTATGGCTGTTCTCATGGC	IDT
oYD998	PCR primer	PCR 1F for *gsa-1*	GAGTTCTACGATCACATTCT	IDT

oYD913	PCR primer	PCR 2F for *reporter*	gggagaccggcagatctAACTTCAAAATTAGACACAA CATTG	IDT
oYD999	PCR primer	PCR2F for *gsa -1*	CACTTGCTGGAAAGACAAGG	IDT
oYD1003	PCR primer	PCR2R for *reporter* and *gsa-1*	GGCGTCGCCATATTCTACTT	IDT

Table S3, RNA FISH probes, Related to STAR Methods

Sequence	Dye	Source
aaaagttcttctcctttact	cy5	IDT
caagaattgggacaactcca	cy5	IDT
cccattaacatcaccatcta	cy5	IDT
cctctccactgacagaaaat	cy5	IDT
gtaagttttccgtatgttgc	cy5	IDT
gtagttttccagtagtgcaa	cy5	IDT
acaagtgttggccatggaac	cy5	IDT
ggtatctcgagaagcattga	cy5	IDT
tcatgccgtttcatatgatc	cy5	IDT
gggcatggcactcttgaaaa	cy5	IDT
ttctttcctgtacataacct	cy5	IDT
gttcccgtcatctttgaaaa	cy5	IDT
tgacttcagcacgtgtcttg	cy5	IDT
taacaagggtatcaccttca	cy5	IDT
ataccttttaactcgattct	cy5	IDT
gtgtccaagaatgtttccat	cy5	IDT
gtgagttatagttgtattcc	cy5	IDT
gtctgccatgatgtatacat	cy5	IDT
ctttgattccattcttttgt	cy5	IDT
ccatcttcaatgttgtgtct	cy5	IDT
atggtctgctagttgaacgc	cy5	IDT
cgccaattggagtattttgt	cy5	IDT
gtctggtaaaaggacagggc	cy5	IDT
aagggcagattgtgtggaca	cy5	IDT
tcttttcgttgggatctttc	cy5	IDT
tcaagaaggaccatgtggtc	cy5	IDT
aatcccagcagctgttacaa	cy5	IDT
tatagttcatccatgccatg	cy5	IDT

Table S4, DNA FISH probes, Related to STAR Methods

Sequence	Modification	Source
**Detection oligo**		
CACACGCTCTTCCGTTCTATGCGACGTCGGTG	Cy5	IDT
**Primary oligos**		
CACCGACGTCGCATAGAACGGAAGAGCGTGTGTCGAGTGGAACAAGCAATGTGCTCTTTCACTGAGTTGTCATCACCA		IDT
CACCGACGTCGCATAGAACGGAAGAGCGTGTGCGTGGCAAACAGGGCTTCAAGGATATTGTCGGCTCTTTTGTTTCA		IDT
CACCGACGTCGCATAGAACGGAAGAGCGTGTGCTCCAAGTACTTCATTCGATTCAGTGACCGTAAAGATGGAGCCGGC		IDT
CACCGACGTCGCATAGAACGGAAGAGCGTGTGAAACGATCGAGAGTTGACACTCGTGACAGACAGGGAAGAAACTCGT		IDT
CACCGACGTCGCATAGAACGGAAGAGCGTGTGTCCGAAAAGTGAGCCTCGATGTAGCTAGCACAGTTCTCATCAAGGA		IDT
CACCGACGTCGCATAGAACGGAAGAGCGTGTGTCCGGAAACAGAAGGTTTTGAACCACCAAGCAGAAGTCGATGC		IDT
CACCGACGTCGCATAGAACGGAAGAGCGTGTGAGACGCTTATATGACATTGCCAATGTCCTGGTTGCTCTTGGGC		IDT
CACCGACGTCGCATAGAACGGAAGAGCGTGTGAAATCCCGCTTTTCGTTTACTGTGGTCCTGAACCTGATCAAACCGG		IDT
CACCGACGTCGCATAGAACGGAAGAGCGTGTGAAAAGCACAGACTGACAAAATTGTGCAACAAATCGCCGGATTCGGG		IDT
CACCGACGTCGCATAGAACGGAAGAGCGTGTGTAATTTGTTTATGTTCGCAGAGGTGGTTACTGCTGAGGCTGCAGCT		IDT
CACCGACGTCGCATAGAACGGAAGAGCGTGTGATGCACCACTTACATCTTCACAGCATCCAATGACATCCAGGCTTCC		IDT
CACCGACGTCGCATAGAACGGAAGAGCGTGTGACCTCTTCCAATGGCTCCTCTTCAACTTCCCAAGCTTCCAGA		IDT
CACCGACGTCGCATAGAACGGAAGAGCGTGTGGATTCCAAAGCCACAGGCTCAAAAGCAGTTTACATTCCCAGCAACG		IDT
CACCGACGTCGCATAGAACGGAAGAGCGTGTGATTTCCCTGACTATACACCTGTGCAATCGACAATACGCCCACTCG		IDT
CACCGACGTCGCATAGAACGGAAGAGCGTGTGAATCAGAATACGTTCGAGCACACGACATCTTCCGCATTCCAGGT		IDT
CACCGACGTCGCATAGAACGGAAGAGCGTGTGTCCAACGTCTTCTGTCGTCATCTATGTGTGTATGTGTGAGGCGACA		IDT
CACCGACGTCGCATAGAACGGAAGAGCGTGTGGACGGTGTGTGATTGCGTAACGGAAGAAGTTTGCGCAATTCG		IDT
CACCGACGTCGCATAGAACGGAAGAGCGTGTGATGGTTAGAGTGTAGGGGCAGACGAGGAGAAGAGCATCTTTTCACA		IDT
CACCGACGTCGCATAGAACGGAAGAGCGTGTGGGTGAGAAAACTTTTCGACGATGGCGTGACTTGATAGAGGTAGGCG		IDT
CACCGACGTCGCATAGAACGGAAGAGCGTGTGCTGCTGAGAAGATTAGATCTGCGCCCTTGCCTTCACCACG		IDT
CACCGACGTCGCATAGAACGGAAGAGCGTGTGTTTTGGATTGGCGATGGAAATAGTTGGGTGAAGATCGGGTTACCGT		IDT
CACCGACGTCGCATAGAACGGAAGAGCGTGTGCTACTTTGCGTGCACATGACCTTCATCCCTCTGGATAGAACCATGG		IDT
CACCGACGTCGCATAGAACGGAAGAGCGTGTGTGATCGAAGATGTTCCGAAGAGAAGAAGAGGGTGGCTGATTGCAC		IDT
CACCGACGTCGCATAGAACGGAAGAGCGTGTGAAGCACAAAGAGAGAAAGGTTCTGAAGAGCACTGGGCATTGCG		IDT

CACCGACGTCGCATAGAACGGAAGAGCGTGTGCAACTTGCGC GCAGATTGGCCGGACGAAAGAACGCGGTTG		IDT
CACCGACGTCGCATAGAACGGAAGAGCGTGTGTTCGCCTTCAC CGATTTTCAAATTTTGGCGCATTTTGTCACCTTGC		IDT
CACCGACGTCGCATAGAACGGAAGAGCGTGTGACAACGTAAGA CTAGAAATTTCGAAAAGGGACGCCTCTTCGCTCGC		IDT
CACCGACGTCGCATAGAACGGAAGAGCGTGTGAAAGCGAGAA ATTTGCAAATGGCAACAACAATTGGCGCAAGACACT		IDT
CACCGACGTCGCATAGAACGGAAGAGCGTGTGAAGGGTGACA CAAGAGAACGAGATGAAGATTGATGGCCTTGGAACG		IDT
CACCGACGTCGCATAGAACGGAAGAGCGTGTGAGTAAGAAATG ATCCCGCACGTAGTGATTCCGCGTTACAAGCCA		IDT
CACCGACGTCGCATAGAACGGAAGAGCGTGTGTTTTGTAGATC GACAACAAGGCCTCCACTTCTTCATCGATGGCCTC		IDT
CACCGACGTCGCATAGAACGGAAGAGCGTGTGTTTTCTTTGAA AGCCGTGCGTGACATGGGCCACGGATTGT		IDT
CACCGACGTCGCATAGAACGGAAGAGCGTGTGAAAATTTGGCA CTTGCTCGAGAGAAACAACGCATTAAGAGGGCTGC		IDT
CACCGACGTCGCATAGAACGGAAGAGCGTGTGTTTTGTAGGTC GACAACGAGGCTGATCGAATAGATGAAGGTGCCG		IDT
CACCGACGTCGCATAGAACGGAAGAGCGTGTGCGAGTTCCAAA TGGCTCTACCTGATTCTTGGCATGCATTTGCCAAT		IDT
CACCGACGTCGCATAGAACGGAAGAGCGTGTGGGCTGACAAG CAGGAACTATCCAAGGAATGCAGCACAAAACTGAGT		IDT
CACCGACGTCGCATAGAACGGAAGAGCGTGTGAGAGATGTTCC GCCAGGCCACAGCAGAAGTGAGTTGATTAAAATCA		IDT
CACCGACGTCGCATAGAACGGAAGAGCGTGTGTGATCCACAG CTCGATCCACCACTCTACAATGCTTGCAAGAGTACT		IDT
CACCGACGTCGCATAGAACGGAAGAGCGTGTGACATTGACTTC TCTCCACGTTGTTCTGCCGAAGTTTCGAGAGCT		IDT
CACCGACGTCGCATAGAACGGAAGAGCGTGTGCGACCACAGC TTTTGACTTCGTGCAGGAAGGAAGCTGAACAATAGT		IDT
CACCGACGTCGCATAGAACGGAAGAGCGTGTGGCCAAAGGAT TTGACAGACGACTGCAAGAAACAACTCAAAGTGGCT		IDT
CACCGACGTCGCATAGAACGGAAGAGCGTGTGGCTGTCTTTCG GAAGTCCGATTCGAGCACAAGGTTTTGGGATC		IDT
CACCGACGTCGCATAGAACGGAAGAGCGTGTGGGCCTGCAAG CCAGTTATTTCCACTCATTGTGCGCAATTTGCTAAT		IDT
CACCGACGTCGCATAGAACGGAAGAGCGTGTGGTGAAGCGCT CTCAGAATTCTGTTCCCATAACGTCAAACCATCGGA		IDT
CACCGACGTCGCATAGAACGGAAGAGCGTGTGCGCGCCGTCC GTGTCAATTTAATTCCGGAAATTGAGGATTCATGTC		IDT
CACCGACGTCGCATAGAACGGAAGAGCGTGTGCGCAAGTGCT CGCCTGTCTCTACAGAAGTGCATATGACGAACAAAA		IDT
CACCGACGTCGCATAGAACGGAAGAGCGTGTGTGTGCCCAAG ACGCAGTTTCAGAAACTGCTACTTTAACATGCCTCA		IDT
CACCGACGTCGCATAGAACGGAAGAGCGTGTGCCGAAGGAAG AACATTGCACTGTTTGATGGAGCATGCTGAATCCAG		IDT
CACCGACGTCGCATAGAACGGAAGAGCGTGTGTCCGAATGGC TCCAGAGCTTGTACTCAACTGTGCACAAGAAATTGA		IDT
CACCGACGTCGCATAGAACGGAAGAGCGTGTGCAGCCGCTCA TTTCCATCTGGCATGGATCCTTTTGTGTCTTGAAA		IDT
